# Shieldin and CST co-orchestrate DNA polymerase-dependent tailed-end joining reactions independently of 53BP1-governed repair pathway choice

**DOI:** 10.1038/s41594-024-01381-9

**Published:** 2024-09-03

**Authors:** Ashleigh King, Pia I. Reichl, Jean S. Metson, Robert Parker, Daniella Munro, Catarina Oliveira, Lucia Sommerova, Jordan R. Becker, Daniel Biggs, Chris Preece, Benjamin Davies, J. Ross Chapman

**Affiliations:** 1 Genome Integrity laboratory, Medical Research Council Molecular Haematology Unit, MRC Weatherall Institute of Molecular Medicine, Radcliffe Department of Medicine, University of Oxford, Oxford, UK; 2 Centre for Immuno-Oncology, Nuffield Department of Medicine, University of Oxford, Oxford, UK; 3 Wellcome Centre for Human Genetics, University of Oxford, Oxford, UK; 4 Francis Crick Institute, London, UK

**Keywords:** Double-strand DNA breaks, Non-homologous-end joining, Immunology, DNA damage and repair

## Abstract

Tumor suppressor p53-binding protein 1 (53BP1) regulates DNA end joining in lymphocytes, diversifying immune antigen receptors. This involves nucleosome-bound 53BP1 at DNA double-stranded breaks (DSBs) recruiting Rap1-interacting factor 1 homolog (RIF1) and shieldin, a poorly understood DNA-binding complex. The 53BP1–RIF1–shieldin axis is pathological in *BRCA1*-mutated cancers, blocking homologous recombination (HR) and driving illegitimate nonhomologous end joining (NHEJ). However, how this axis regulates DNA end joining and HR suppression remains unresolved. We investigated shieldin and its interplay with the Ctc1–Stn1–Ten1 (CST) complex, which was recently implicated downstream of 53BP1. Immunophenotypically, mice lacking shieldin or CST are equivalent, with class-switch recombination coreliant on both complexes. Ataxia-telangiectasia mutated kinase-dependent DNA damage signaling underpins this cooperation, inducing physical interactions between these complexes that reveal shieldin as a DSB-responsive CST adaptor. Furthermore, DNA polymerase ζ functions downstream of shieldin, establishing DNA fill-in synthesis as the physiological function of shieldin–CST. Lastly, we demonstrate that 53BP1 suppresses HR and promotes NHEJ in *BRCA1*-deficient mice and cells independently of shieldin. These findings showcase the versatility of the 53BP1 pathway, achieved through the collaboration of chromatin-bound 53BP1 complexes and DNA end-processing effector proteins.

## Abstract (web summary)

Here, using mouse genetics, biochemistry and cell-based experiments, the authors reveal that shieldin primarily catalyzes DNA priming and polymerase-dependent fill-in synthesis at 5′ recessed DNA ends during the joining of activation-induced cytidine deaminase-dependent DNA breaks.

## Main

DNA double-strand breaks (DSBs) are highly toxic and must be repaired accurately to counteract the threat of human disease and oncogenic mutations^1^. Paradoxically, mutagenic DSB repair by nonhomologous end joining (NHEJ) is favored in developing and antigen-stimulated lymphocytes, where it mediates deletional recombination events that diversify the antigenic specificity and function of B and T cell receptors^2^. To cope with this intrinsic discrepancy in desired DNA repair outcome between different cellular contexts, cells have evolved complex regulatory systems that maintain an appropriate equilibrium between competing DNA repair pathways, ensuring that DNA breaks are appropriately resolved^3^.

The generation of functional B and T cell receptors by the mammalian adaptive immune system relies on two programmed gene rearrangement mechanisms: V(D)J recombination and class-switch recombination (CSR)^2^. Both mechanisms rely on intragenic deletional recombination events, which are mediated through the ligation of interspaced DNA ends using NHEJ pathways. Because of the distinct biochemical mechanisms involved in generating DSBs during V(D)J recombination and CSR, the resulting DNA ends have different structural properties, necessitating distinct DNA end-processing and ligation mechanisms^4^.

DSB induction during V(D)J recombination occurs during the development of B and T cells in the bone marrow and thymus, respectively. The process is orchestrated by recombination-activating gene enzymes 1 and 2 (RAG1/2), which cleave V, D and J segment-flanking recombination signal sequences (RSSs), resulting in pairs of blunt signal and covalently sealed hair-pinned coding ends. Hairpin opening is essential before coding joins can occur, a step facilitated by the Artemis endonuclease upon its activation by the DNA-dependent protein kinase (DNA-PK) complex. This generates DNA ends that are strictly joined by classical NHEJ (c-NHEJ) proteins including Ku (Ku70 and Ku80), X-ray repair cross-complementing protein 4 (XRCC4) and DNA ligase 4 (LIG4), with a more modest reliance on XRCC4-like factor (XLF)^2^.

In contrast, CSR in mature peripheral lymphocytes involves DSB generation between antibody isotype-encoding constant (*C*) gene segments of the immunoglobulin heavy chain locus (*igh)*. This process is initiated by activation-induced cytidine deaminase (AID), which introduces clusters of U•G mismatches in actively transcribed switch *(S)* introns. These mismatches are then processed by base excision repair (BER) and mismatch repair (MMR) machineries, ultimately leading to the conversion of single-stranded DNA (ssDNA) gaps into DSBs with single-stranded tailed termini. Unlike RAG1/2-induced DSBs, AID-dependent DSBs during CSR can be repaired through both c-NHEJ and alternative end joining (a-EJ) pathways, with the tumor suppressor p53-binding protein 1 (53BP1) pathway proteins also having a critical role^4^.

The 53BP1 pathway is composed of nucleosomal and DNA-binding protein components. The most upstream component is 53BP1, a chromatin reader protein scaffold that interacts with the DSB-responsive ubiquitinated Lys15 on nucleosomal H2A type histones (H2AK15ub) and ubiquitous histone H4 methylation at Lys20 (H4K20me1/2)^5,6^. This interaction leads to the formation of extensive 53BP1 chromatin-bound domains at DSB sites. Downstream of 53BP1, the 53BP1-binding protein Rap1-interacting factor 1 homolog (RIF1) bridges interactions between Ataxia-telangiectasia mutated kinase (ATM)-phosphorylated motifs in 53BP1 and the SHLD3 subunit of shieldin^7,8^, a complex bound to ssDNA that is thought to protect DNA ends^9^. Studies using genetically engineered mouse models deleted of *53bp1* or shieldin proteins *Rev7* or *Shld1/2* present with near-complete deficiencies in CSR. By contrast, only *53bp1*^*−/−*^ mice exhibit V(D)J recombination defects, which lead to mild B and T cell lymphopenia^10–13^.

The importance of the 53BP1 pathway extends to *BRCA1*-mutated cancers. In normal cells, the BRCA1 (breast cancer type 1 susceptibility protein)–BARD1 (BRCA1-associated RING domain 1) heterodimer binds to a related combinatorial chromatin signature comprising H2AK15ub and unmethylated Lys20 on histone H4 (a postreplicative modification state), excluding 53BP1 from DNA damage sites and directing homologous recombination (HR)^14,15^. However, in *BRCA1-*deficient cells, uninhibited 53BP1–chromatin complexes prevent HR and promote NHEJ-mediated genome rearrangements, leading to genomic instability^16,17^. Consequently, *BRCA1*-deficient cells and tumors are hypersensitive to poly(ADP-ribose) polymerase (Pol) inhibitors (PARPis), which exploit the HR defect caused by 53BP1 (ref. ^16^). Conversely, *53BP1* deletion is able to restore PARPi resistance to *BRCA1-*mutant cells^18^ and can even rescue viability in *Brca1-*null mice^19,20^.

Genetic screens aimed at elucidating PARPi resistance mechanisms in *BRCA1*-deficient cells were instrumental in revealing the 53BP1 pathway biology. Shieldin genes were discovered through screens investigating PARPi-induced toxicity in *BRCA1*-deficient cells^21–23^ and the Pol α–primase accessory complex proteins Ctc1–Stn1–Ten1 (CST) similarly emerged as putative 53BP1 pathway effectors^24^. Concurrent hypothesis-guided studies also linked the activities of shieldin and CST to pathological 53BP1 pathway function in *BRCA1-*mutant cells^11,25–28^ and highlighted CST-directed Pol α–primase-dependent DNA fill-in synthesis as a key intermediary step^27,29,30^.

This research has ignited debates regarding the precise role of shieldin, particularly whether its primary function involves blocking nucleolytic resection or facilitating fill-in synthesis at DNA ends^4,31^. The importance of effector interactions in the 53BP1 pathway during physiological antigen receptor recombination mechanisms, as well as their impact on HR defects and DNA joining events in *BRCA1*-deficient cells, remains to be fully understood. To address these questions, we conducted a comprehensive analysis using genetically engineered mouse models. Our findings demonstrate that the 53BP1 pathway contributes substantially to DNA end joining during antigen receptor recombination and in cancer cells in the absence of shieldin and CST. We also demonstrate a strict interdependence between shieldin and CST during CSR and an epistatic relationship with DNA Pol ζ, solidifying fill-in synthesis as a primary shieldin-dependent DNA repair function. Importantly, using *BRCA1-*deficient mice and cellular models, we establish that HR suppression relies on 53BP1 but not shieldin. Altogether, our results showcase remarkable resilience in the 53BP1 pathway, achieved through a division of labor between chromatin-bound 53BP1 complexes and their downstream DNA-bound effectors.

## Results

### Shieldin and CST equivalency during lymphocyte development

**Fig. 1:**
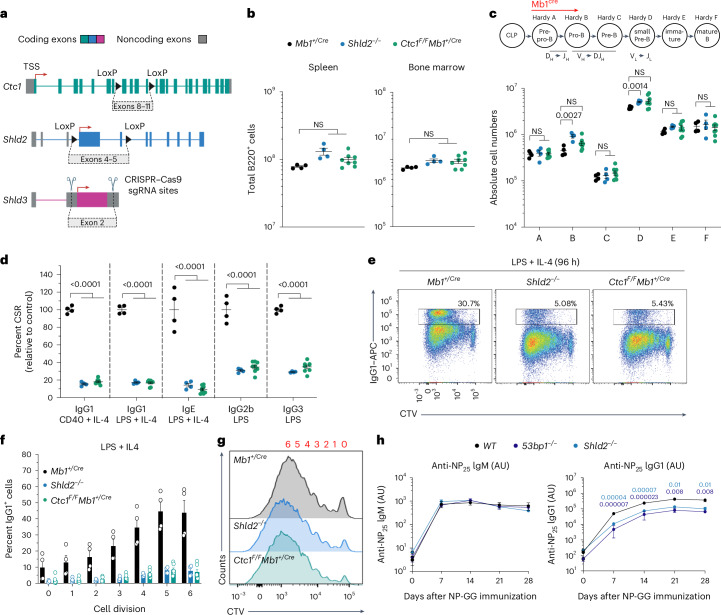
Equivalent immunodeficiencies in mice lacking shieldin and CST. **a**, Schematic representation of the *Shld2* and *Shld3* (knockout) and *Ctc1* (conditional) alleles generated in this study. TSS, transcription start site. **b**, Absolute numbers of B220^+^ B cells in the bone marrow (one femur and one tibia) and spleen (*n* = 4–8 mice per genotype, where each data point is a single mouse). Significance was determined by an unpaired two tailed *t*-test (mean ± s.e.m.). NS, not significant. **c**, Absolute numbers of B cell precursors (Hardy fraction A, B220^+^CD43^+^BP1^−^CD24^−^; Hardy fraction B, B220^+^CD43^+^BP1^−^CD24^+^; Hardy fraction C, B220^+^CD43^+^BP1^+^CD24^+^; Hardy fraction D, B220^+^CD43^−^IgM^−^IgD^−^; Hardy fraction E, B220^+^CD43^−^IgM^+^IgD^−^; Hardy fraction F, B220^+^CD43^−^IgM^+^IgD^+^) in the bone marrow (one femur and one tibia) (*n* = 4–8 mice per genotype, where each data point is a single mouse). Significance was determined by an unpaired two tailed *t*-test (mean ± s.e.m.). **d**, Splenic B cells cultured with the indicated stimuli (96 h) and stained for surface IgG1, IgE, IgG2b or IgG3 (*n* = 4–7 mice per genotype, where each data point is a single mouse). CSR 100%, mean immunoglobulin isotype switch frequency of two control animals in each experiment. Significance was determined by a two-way analysis of variance (ANOVA) with Tukey’s correction (mean ± s.e.m.). **e**, CTV-labeled splenic B cells were stimulated as indicated and stained for surface IgG1 after 96 h. Representative data, *n* > 6 mice. **f**, IgG1^+^ B cells as a proportion of total B cells (%) for each cell generation as determined by CTV staining and proliferation-associated dye dilution (*n* = 4–6 mice per genotype, where each data point is a single mouse; mean ± s.e.m.). **g**, CTV dilution in purified B cells cultured in the presence of LPS and IL-4 for 96 h. Representative data, *n* > 6 mice. **h**, NP-specific serum IgM (left) and IgG1 (right) at indicated times after NP-CGG immunization. Representative data, *n* = 2 independent experiments, each with four mice. Significance was determined by an unpaired two tailed *t*-test (mean ± s.e.m.). Gating strategies for the above flow cytometry data are provided in Supplementary Fig. 1. Source data

**Extended Data Fig. 1:**
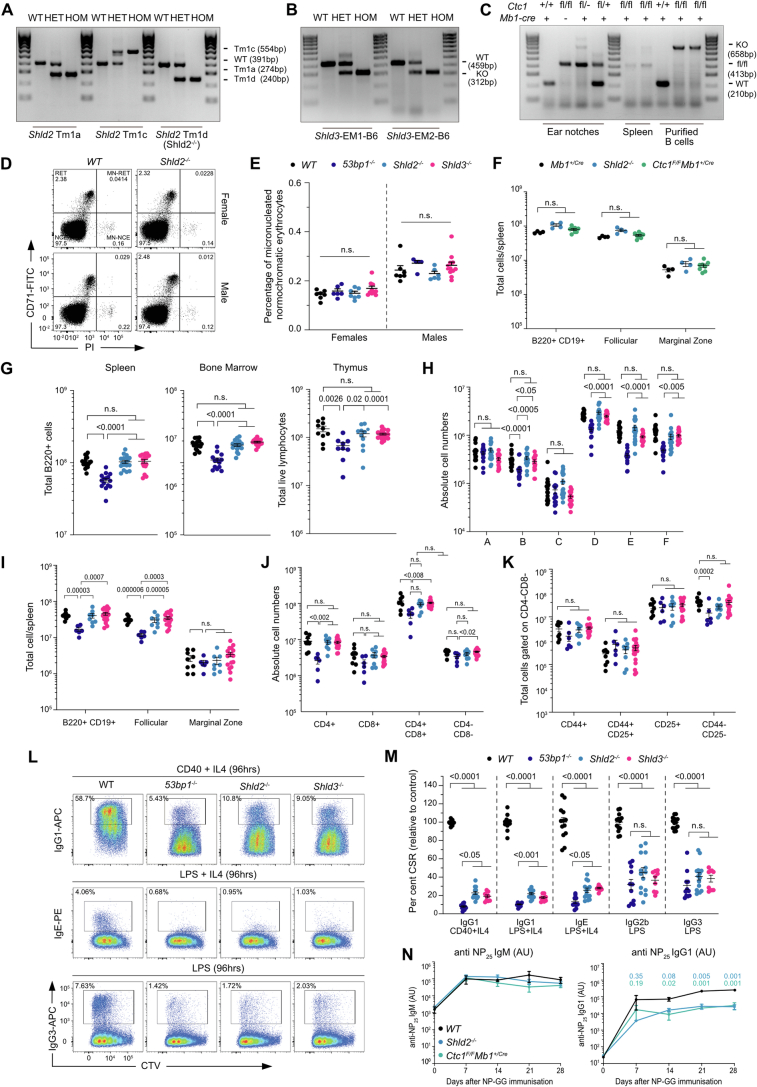
Equivalent immunodeficiencies in mice lacking shieldin and CST. **a)** Representative image of PCR amplicons from genomic DNA obtained by ear biopsies from mice of the indicated genotype. Bands of different size correspond to the *Shld2*^*tm1a*^ allele (274 bp), *Shld2*^*tm1c*^ allele (554 bp), *Shld2*^*tm1d*^ allele (240 bp) and *WT* allele (391 bp). n = >20 mice per genotype. **b)** Representative image of PCR amplicons from genomic DNA obtained by ear biopsies from mice of the indicated genotype. Bands of different size correspond to the *Shld3*^*EM1-B6*^ allele (left) or *Shld3*^*EM2-B6*^ allele (right) (~312 bp) and *WT* allele (459 bp). n = >20 mice per genotype. **c)** Representative image of PCR amplicons from genomic DNA obtained by ear biopsies (left), splenic b cells or purified splenic b cells (right) from mice of the indicated genotype. Bands of different size correspond to the *Ctc1*^*fl*^ allele (413 bp), *Ctc1*^*−/−*^ allele (658 bp) and *WT* allele (210 bp). n = >20 mice per genotype except for purified B cells; n = 2 independent experiments each with 2-3 mice. **d)** Peripheral blood stained as indicated. Reticulocytes (RET), micronucleated reticulocytes (MN-RET), normochromatic erythrocytes (NCE) and micronucleated normochromatic erythrocytes (MN-NCE). Representative data, n ³ 5 mice of each sex per genotype. **e)** MN-NCE as a percentage of total NCEs from tail vein blood of mice at 8-12 weeks. n = 5-12 mice of each sex per genotype, where each data point is a single mouse. Significance was determined by unpaired two tailed *t*-test, Mean ± SEM. **f)** Flow cytometric sub-classification of nature splenic b cell fractions. n = 4-8 mice per genotype, where each data point is a single mouse. Significance was determined by unpaired two tailed *t*-test, Mean±SEM. **g)** Absolute numbers of B220^+^ B cells in the bone marrow (one femur and one tibia) and spleen and live lymphocytes in the thymus. n = 10-18 mice per genotype, where each data point is a single mouse. Significance was determined by unpaired two tailed *t*-test, Mean ± SEM. **h)** Absolute numbers of B cell precursors in the bone marrow (one femur and one tibia). n = 15-19 mice per genotype, where each data point is a single mouse. Significance was determined by unpaired two tailed *t*-test, Mean±SEM. **i)** Flow cytometric sub-classification of mature splenic b cell fractions. n = 6-17 mice per genotype, where each data point is a single mouse. Significance was determined by unpaired two tailed *t*-test, Mean ± SEM. **j)** Absolute numbers of thymic T-cells, n = 9-17 mice per genotype, where each data point is a single mouse. Mean ± SEM. **k)** Absolute numbers of T cell precursors in double negative (CD4- CD8-) in the thymus, n = 9-17 mice per genotype, where each data point is a single mouse. Mean ± SEM. **l)** CTV-labelled purified B cells were stimulated as indicated and stained for surface IgG/IgE on day 4. Representative of *n* > 6 experiments. **m)** Splenic B cells cultured with the indicated stimuli (96 h) and stained for surface IgG1, IgE, IgG2b or IgG3. *n* = 11-14 mice per genotype. CSR 100%, mean immunoglobulin isotype switch frequency of 2 control animals in each experiment. Significance was determined by two-way ANOVA with Tukey’s correction. Mean ± SEM. **n)** NP-specific serum IgM (left) and IgG1 (right) at indicated times after NP-CGG immunization. AU, arbitrary units. Representative data, n = 2 independent experiments, each with 2-3 mice. Significance was determined by unpaired two tailed *t*-test. Mean ± SEM. Source data

To understand the extent to which shieldin–CST cooperation supports physiological 53BP1 pathway function, we generated new mouse strains deleted for genes encoding core proteins in each complex in an inbred C57BL/6 background. Knockout mice deleted for critical exons 4–5 in *Shld2* or exon 2 in *Shld3*, which comprises its entire coding sequences (hereafter referred to as *Shld2*^*−*/*−*^ and *Shld3*^*−*/*−*^ mice; Fig. 1a and Extended Data Fig. 1a,b), were viable, healthy, fertile and born at expected Mendelian frequencies. Moreover, genomic instability in 8–12-week-old *Shld2*^*−*/*−*^ and *Shld3*^*−*/*−*^ mice did not exceed that seen in aged-matched cohorts of wild-type (WT) or *53bp1*^*−/−*^ mice, as defined by levels of micronuclei in erythrocytes (Extended Data Fig. 1d,e), a proxy for systemic genomic instability^32^.

Mutations affecting the CST gene *CTC1* in humans cause Coats plus syndrome, an autosomal recessive disorder characterized by retinal telangiectasia, intracranial calcifications, osteopenia, gastrointestinal bleeding and, in severe cases, normocytic anaemia reflecting a degree of bone marrow failure^33^. Similarly, hematopoietic stem cell (HSC) exhaustion characterizes mice homozygous for *Ctc1* gene deletions, leading to complete bone marrow failure and perinatal lethality^34^. International Mouse Phenotyping Consortium (IMPC)^35^ phenotyping of mice homozygous for a knockout-first conditional (ready) allele in *Ctc1 (Ctc1*^*tm1a(KOMP)Wtsi*^) also discovered perinatal lethality in *Ctc1-*deleted mice^36^. Thus, *Ctc1* essentiality in murine hematopoiesis prompted us to investigate the effect of *Ctc1* deletion in the B lymphocyte lineage, where the 53BP1 pathway supports normal development and programmed genome diversification. To this end, we generated conditional *Ctc1*^*tm1c/tm1c*^ *Mb1*^+/Cre^ mice (hereafter referred to as *Ctc1*^*F/F*^ *Mb1*^+/Cre^ mice), in which *Cre* expression from the *Mb1-Cre* transgene mediated complete deletion of *Ctc1* critical exons 8–11 in early B cell progenitors (Fig. 1a and Extended Data Fig. 1c). To our surprise, CST deficiency was well tolerated in developing and differentiating B lymphocytes. *Ctc1*^*F/F*^ *Mb1*^+/Cre^ mice showed normal cell frequencies across all stages of lineage development in the bone marrow and B cell maturation in the spleen (Fig. 1b,c and Extended Data Fig. 1f). The normal development of *Ctc1*^*∆/∆*^ B cells aligned with the phenotypes of *Shld2*^*−*/*−*^ and *Shld3*^*−*/*−*^ mice, which similarly fostered normal B and T cell lineage development (Extended Data Fig. 1g–k). Normal lymphocyte development in *shieldin-*knockout mice is because of proficiency in V(D)J recombination, as demonstrated in our previous characterization of mice deleted of the shieldin gene *Rev7* (ref. ^11^) and more recent concordant results in *Shld1*–*Shld2-*mutant mice^12,13^. Thus, neither CST nor shieldin contributes to lymphocyte development, where 53BP1 supports efficient DNA end joining during V(D)J recombination^10,11^.

### Shieldin–CST cooperation underpins productive CSR

We next sought to define the importance of shieldin–CST interplay for the joining of AID-dependent DSBs during CSR, a process entirely dependent on 53BP1 that also requires the *Rev7* and shieldin proteins^11–13^. To this end, we stimulated cultures of mature B splenocytes from *Ctc1*^*F/F*^ *Mb1*^+/Cre^, *Shld2*^*−*/*−*^ and *Shld3*^*−*/*−*^ mice and analyzed their ability to support antibody isotype switching in vitro. Strikingly, ex vivo stimulation of mature splenic B cells from *Ctc1*^*F/F*^ *Mb1*^+/Cre^ mice revealed severe (>5-fold) defects in CSR across all analyzed IG isotypes, fully recapitulating the magnitude of defects presented in *Shld2-*deficient B cells analyzed in parallel (Fig. 1d,e). Defective class switching in *Shld2*-deficient and *Ctc1*-deficient B cells did not correlate to defects or differences in cell proliferation or survival (Fig. 1f,g). Despite this, B cells from *Ctc1*^*F/F*^ *Mb1*^+/Cre^ mice and both *shieldin*-knockout mouse strains supported higher class-switching frequencies than those from *53bp1*^*−/−*^ mice, where CSR was reduced >10-fold relative to WT (Fig. 1d,e and Extended Data Fig. 1l,m). These differences also held true in vivo in mice immunized with the model antigen NP-CGG (3-hydroxy-nitrophenyl acetyl coupled to chicken γ-globulin). Following immunization, serum titers of NP-specific IgG1 were strongly attenuated in *Shld2*^*−/−*^*, 53bp1*^*−/−*^ and *Ctc1*^*F/F*^ *Mb1*^+/Cre^ mice relative to WT controls, despite exhibiting WT IgM-mediated antigen responses as expected (Fig. 1h and Extended Data Fig. 1n). However, antigen-specific IgG1 consistently accumulated to higher levels in *Shld2*^*−/−*^ mice than in *53bp1*^*−/−*^ mice at all time points following immunization (Fig. 1h). By contrast, NP-specific IgG1 titers accumulated at equivalent levels following immunization in *Ctc1*^*F/F*^ *Mb1*^+/Cre^ and *Shld2*^*−/−*^ mice (Extended Data Fig. 1n). Considered together, our results show that the 53BP1 pathway retains some capacity to support CSR in the absence of shieldin and CST. The fact that B lymphocyte development and differentiation capacities were found to be identical in *Ctc1*^*F/F*^ *Mb1*^+/Cre^*, Shld2*^*−/−*^ and *Shld3*^*−/−*^ mice prompted us to next investigate the mechanistic basis of shieldin’s coordination with CST and downstream factors in CSR.

### ATM promotes shieldin–CST interactions during DSB repair

**Fig. 2:**
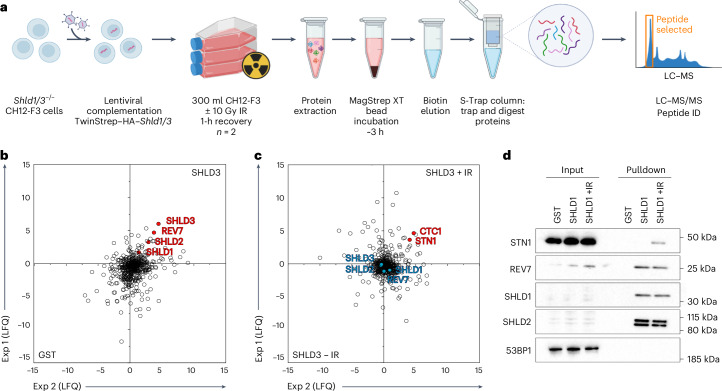
DNA damage induces interactions between shieldin and CST. **a**, Schematic depicting shieldin purification and proteomic elucidation strategy. Briefly, *Shld1/3*-knockout CH12-F3 cell lines were lentivirally transduced with TwinStrep–HA–Shld1/3 espression transgenes. First, 300-ml suspension cultures were either mock-treated or irradiated (10 Gy) and lysates were prepared following a 1-h recovery. Shieldin complexes captured from lysates on MagStrep-XT resin were eluted in biotin, captured and tryptic-digested on S-Trap columns, with resulting peptides analyzed by LC–MS/MS. The schematic was generated using BioRender.com. **b**, B cell SHLD3 versus control (beads only) interactomes as defined by LC–MS/MS and LFQ. The scatter plot depicts the log_2_ fold enrichment of indicated MagStrep-XT purified complexes across two independent experiments. **c**, Comparison of mock and irradiated B cell SHLD3 interactomes as defined by LC–MS/MS and LFQ. The scatter plot depicts the log_2_ fold enrichment of indicated MagStrep-XT purified complexes across two independent experiments. **d**, Immunoblot analysis of CH12-F3 cell extracts from SHLD3 pulldown experiments. Representative of *n* = *2* independent experiments. Source data

**Extended Data Fig. 2:**
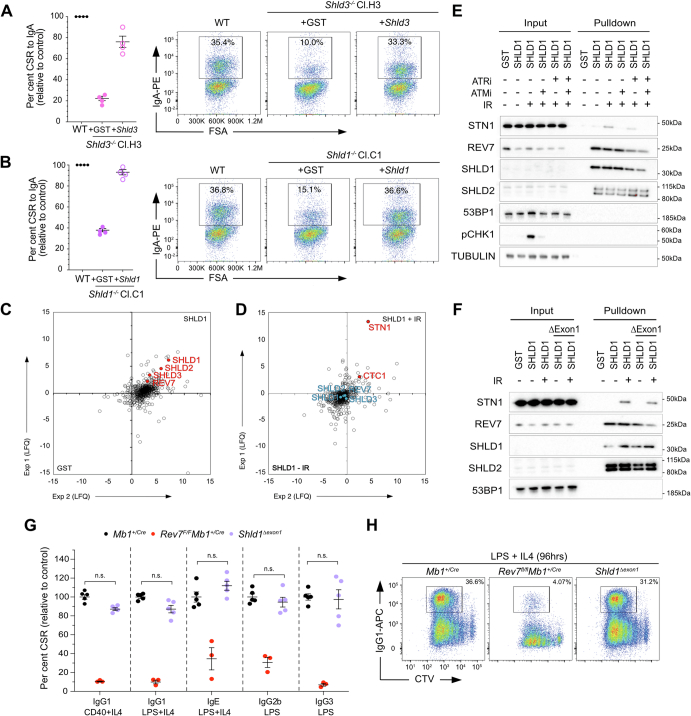
DNA damage induces interactions between shieldin and CST. **a)** IgM-to-IgA CSR frequencies in *Shld3* knockout CH12-F3 cell lines, *n* = 3 independent experiments, where each data point is the average of 3 technical replicates from a single experiment. Mean ± SEM. Representative flow cytometry plots for IgM-to-IgA CSR. Representative data, *n* > 3 independent experiments. **b)** IgM-to-IgA CSR frequencies in *Shld1* knockout CH12-F3 cell lines, *n* = 3 independent experiments, where each data point is the average of 3 technical replicates from a single experiment. Mean ± SEM. Representative flow cytometry plots for IgM-to-IgA CSR. Representative data, *n* > 3 independent experiments. **c)** B cell *Shld1* interactome as defined by LC–MS/MS and LFQ. Scatter plot depicts log_2_ fold-enrichment of indicated TwinStep–immunocomplexes across 2 independent experiments. **d)** B cell *Shld1* IR-dependent interactome as defined by LC–MS/MS and LFQ. Scatter plot depicts log_2_ fold-enrichment of indicated TwinStep–immunocomplexes across 2 independent experiments. **e)** Western blot analysis of CH12-F3 cell extracts from pulldown experiments. Cells were pre-treated with ATM, ATR inhibitor or both for 1 hr prior to irradiation. Representative of n = 2 independent experiments. **f)** Western blot analysis of CH12-F3 cell extracts from pulldown experiments. Representative of n = 2 independent experiments. **g)** Splenic B cells cultured with the indicated stimuli (96 h) and stained for surface IgG1, IgE, IgG2b or IgG3. *n* = 3-5 mice per genotype. CSR 100%, mean immunoglobulin isotype switch frequency of 2 control animals in each experiment. Significance was determined by two-way ANOVA with Tukey’s correction. Mean ± SEM. **h)** CTV-labelled purified B cells were stimulated as indicated and stained for surface IgG on day 4. Representative of *n* = 2 experiments. Source data

To investigate shieldin adaptor functions in DSB repair, we used proteomics to define the shieldin interactome. For this, we introduced TwinStrep-tagged *Shld1* and *Shld3* transgenes into *Shld1*^*−*/*−*^ and *Shld3*^*−*/*−*^ CH12-F3 mouse B cell lymphoma cell lines, respectively, and validated functional complementation at the level of rescued IgM-to-IgA class switching in stimulated cell cultures (Extended Data Fig. 2a,b). Because of shieldin’s extreme low abundance in mammalian cells^26^, its successful purification required lysates prepared from cultures of >6 × 10^9^ cells. Shieldin complexes isolated by Strep-Tactin affinity purification were then subjected to analysis by liquid chromatography–tandem mass spectrometry (LC–MS/MS) over two independent experiments (Fig. 2a).

Highly-efficient purification of shieldin was confirmed by high peptide coverage across all four shieldin proteins (Table 1). Surprisingly, shieldin complexes were notably devoid of significant protein interactors (Fig. 2b). Because interactions between shieldin’s upstream regulators 53BP1 and RIF1 are controlled by DNA damage-dependent phosphorylation^7,8^, our subsequent investigation focused on SHLD1 and SHLD3 (SHLD1/3) complexes obtained from lysates of X-ray-irradiated cells.

**Table 1:** LFQ of LC–MS/MS results

Protein symbol	Unique peptides	Coverage (%)	TS–HA–SHLD1		TS–HA–SHLD3	
			LFQ	LFQ^IR^	LFQ	LFQ^IR^
Rev7 (MAD2L2)	8	32	6.6	−1.0	1.6	0.0
Shld1 (C20orf196)	10	41	5.1	1.2	3.1	−0.3
Shld2 (FAM35A)	46	60	3.4	−0.5	5.3	−0.2
Shld3 (FLJ26957)	14	66	2.6	−0.4	4.2	−0.5
Ctc1	1	–	−3.7	8.7	−0.7	3.9
Stn1	2	–	5.7	2.7	3.4	4.7

Interacting proteins were determined as in Fig. 2a–c (*n* = 2 independent experiments per genotype).

Remarkably, analysis of (TwinStrep–SHLD1/3) purifications under these conditions revealed a discernible enrichment of peptides from CST proteins CTC1 and STN1 in both sets of purifications (Fig. 2c and Extended Data Fig. 2d). The induction of shieldin–CST interactions following DNA damage treatments was validated through immunoblotting of shieldin complex purifications on a smaller scale using anti-STN1 antibodies (Fig. 2d). Additionally, pretreatment of cells with a small-molecule ATM inhibitor (KU55933) but not an ataxia-telangiectasia and Rad3-related protein (ATR) inhibitor (AZD6738) abolished shieldin–CST interactions (Extended Data Fig. 2e).

These findings strongly implicate shieldin as a recruitment platform for the CST complex during DNA repair and point to ATM-dependent DNA damage signaling as the trigger for assembling shieldin–CST complexes at DSB sites. Such a conclusion provides a likely explanation for the equivalent effects of *shieldin* and *CST* gene disruption in murine B cells and, furthermore, implicates Pol α–primase-dependent fill-in synthesis as an intermediary step in the joining of AID-induced DSBs.

### Shieldin-dependent DSB repair involves the reversionless 3-like (REV3L) Pol

During DNA replication, the synthesis of an RNA–DNA primer on the lagging strand by Pol α–primase precedes strand extension by DNA Pol δ^37^. We, therefore, considered that processive fill-in synthesis downstream of CST-directed Pol α–primase priming might likewise rely on Pol δ or an analogous processive DNA Pol. Interestingly, DNA Pol ζ, a translesion synthesis Pol formed of the B-family Pol REV3L in complex with REV7 and Pol δ accessory subunits POLD2 and POLD3 (ref. ^38^), was previously implicated in NHEJ during CSR^39^. This led us to investigate REV3L’s participation in DNA end joining downstream of shieldin–CST.

**Fig. 3:**
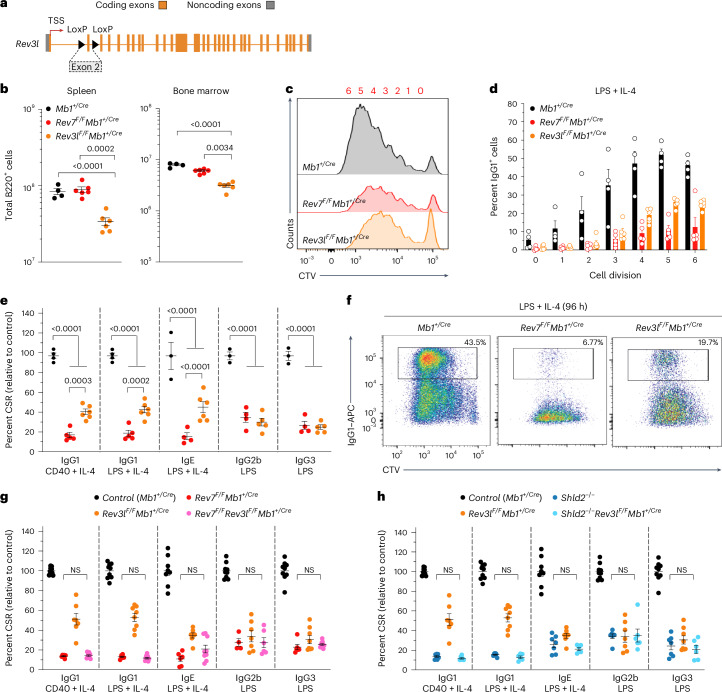
Pol ζ cooperation with shieldin supports NHEJ in CSR. **a**, Schematic representation of the *Rev3l* conditional allele. **b**, Absolute numbers of B220^+^ B cells in the bone marrow (one femur and one tibia) and spleen (*n* = 4–6 mice per genotype, where each data point is a single mouse). Significance was determined by an unpaired two tailed *t*-test (mean ± s.e.m.). **c**, CTV dilution in purified B cells cultured in the presence of LPS and IL-4 for 96 h. Representative data, *n* > 6 mice. **d**, IgG1^+^ B cells as a proportion of total B cells (%) for each cell generation as determined by CTV staining and proliferation-associated dye dilution (*n* = 4–6 mice per genotype, where each data point is a single mouse; mean ± s.e.m.). **e**, Splenic B cells cultured with the indicated stimuli (96 h) and stained for surface IgG1, IgE, IgG2b or IgG3 (*n* = 4–7 mice per genotype, where each data point is a single mouse). CSR 100%, mean immunoglobulin isotype switch frequency of two control animals in each experiment. Significance was determined by a two-way ANOVA with Tukey’s correction (mean ± s.e.m.). **f**, CTV-labeled splenic B cells were stimulated as indicated and stained for surface IgG1 after 96 h. Representative data, *n* > 6 mice. **g**, Splenic B cells cultured with the indicated stimuli (96 h) and stained for surface IgG1, IgE, IgG2b or IgG3 (*n* = 4–7 mice per genotype, where each data point is a single mouse). CSR 100%, mean immunoglobulin isotype switch frequency of two control animals in each experiment. Significance was determined by a two-way ANOVA with Tukey’s correction (mean ± s.e.m.). **h**, Splenic B cells cultured with the indicated stimuli (96 h) and stained for surface IgG1, IgE, IgG2b or IgG3 (*n* = 4–7 mice per genotype, where each data point is a single mouse). CSR 100%, mean immunoglobulin isotype switch frequency of two control animals in each experiment. Significance was determined by a two-way ANOVA with Tukey’s correction (mean ± s.e.m.). Source data

**Extended Data Fig. 3:**
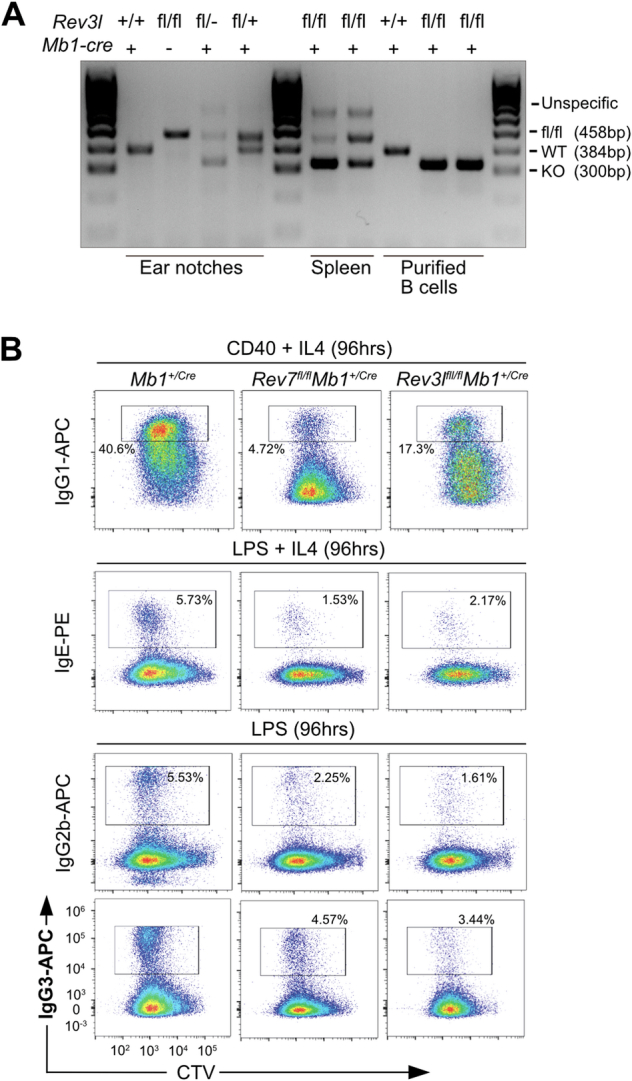
Pol ζ cooperation with shieldin supports NHEJ in CSR. **a)** Representative image of PCR amplicons from genomic DNA obtained by ear biopsies (left), splenic b cells or purified splenic b cells (right) from mice of the indicated genotype. Bands of different size correspond to the *Rev3l*^*fl*^ allele (458 bp), *Rev3l*^*−/−*^ allele (300 bp) and *WT* allele (384 bp). n = >20 mice per genotype except for purified B cells; n = 2 independent experiments each with 2-3 mice. **b)** CTV-labelled purified B cells were stimulated as indicated and stained for surface IgG/IgE on day 4. Representative of *n* > 5 experiments.

*Rev3l* is essential in mice^38^ and prior characterizations of B cell-specific REV3L functions were complicated by mosaicism driven by the out-competition of proliferation-defective *Rev3l-*deleted mature B cells with those that had escaped *CD21-Cre*-mediated recombination^39^. We, thus, opted to breed mice harboring the same *Rev3l*^*tm1(Rsky)*^ conditional knockout allele in combination with the B cell-specific *Mb1-Cre* transgene, which mediates higher-efficiency *Cre-*mediated recombination upstream in B lymphocyte progenitor cells^11,40^. As predicted, *Rev3l* was completely deleted in the B cells of *Rev3l*^*F/F*^ *Mb1*^+/Cre^ mice (Extended Data Fig. 3a), where it resulted in 2–3-fold reductions in absolute B cell frequencies in the bone marrow and spleen (Fig. 3b). Despite this, purified B splenocytes from *Rev3l*^*F/F*^ *Mb1*^+/Cre^ mice were viable in culture and could undergo multiple rounds of cell division upon stimulation ex vivo (Fig. 3c). This allowed us to re-evaluate REV3L’s contribution to CSR. Indeed, *Rev3l*^*F/F*^ *Mb1*^+/Cre^ splenic B cells supported IgM-to-IgG1 class switching but at levels ~40% lower than WT or *Mb1-cre-*positive controls (Fig. 3d–f and Extended Data Fig. 3b). Again, class switching in cell-division-staged *Rev3l-*deficient B cells and their controls confirmed that these reductions were not a consequence of reduced cell proliferation (Fig. 3c,d), a result consistent with REV3L’s likely participation in NHEJ^39^.

To ascertain whether Pol ζ functions downstream of shieldin in CSR, we interbred *Rev3l*^*F/F*^ *Mb1*^+/Cre^ mice with *Shld2-*knockout mice and our previously described B cell conditional *Rev7-*knockout strain^11^, generating mice codeficient for shieldin and REV3L in the B cell lineage. The *ex vivo* stimulation of mature B splenocytes codeleted of *Rev3l* and either shieldin gene confirmed that *Rev3l* loss did not further decrease class switching below that seen in *Rev7* or *Shld2* single-knockout controls (Fig. 3g,h). This genetic epistasis between *Rev3l* and *shieldin* confirms Pol ζ’s participation in shieldin–CST-dependent DNA end-joining reactions. Considering CST’s role in catalyzing Pol α–primase-dependent fill-in synthesis in *BRCA1-*deficient cells and at Cas9-induced DSBs^27,29,30^, our results position Pol ζ as a likely mediator of processive fill-in synthesis downstream of these DNA priming reactions.

### NHEJ-independent REV3L functions support B cell development

**Fig. 4:**
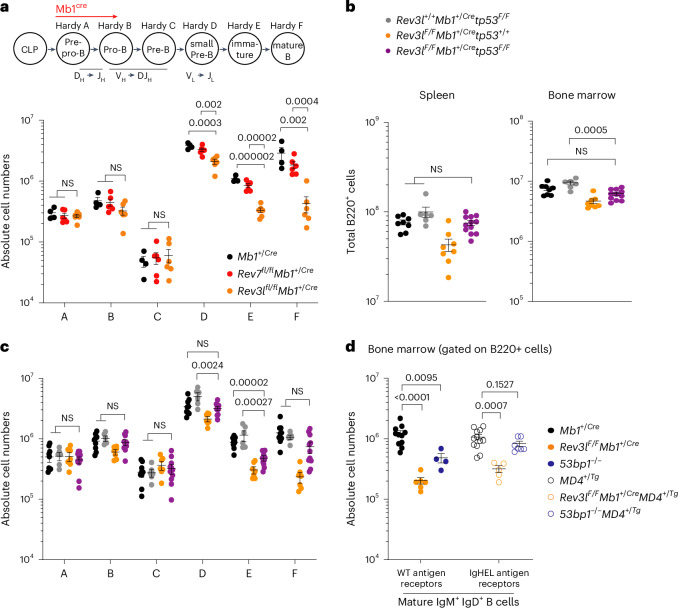
REV7-independent and NHEJ-independent functions of Pol ζ support B cell development. **a**, Absolute numbers of B cell precursors (Hardy fraction A, B220^+^CD43^+^BP1^−^CD24^−^; Hardy fraction B, B220^+^CD43^+^BP1^−^CD24^+^; Hardy fraction C, B220^+^CD43^+^BP1^+^CD24^+^; Hardy fraction D, B220^+^CD43^−^IgM^−^IgD^−^; Hardy fraction E, B220^+^CD43^−^IgM^+^IgD^−^; Hardy fraction F, B220^+^CD43^−^IgM^+^IgD^+^) in the bone marrow (one femur and one tibia) (*n* = 4–6 mice per genotype, where each data point is a single mouse). Significance was determined by an unpaired two tailed *t*-test (mean ± s.e.m.). **b**, Absolute numbers of B220^+^ B cells in the bone marrow (one femur and one tibia) and spleen (*n* = 6–12 mice per genotype, where each data point is a single mouse). Significance was determined by an unpaired two tailed *t*-test (mean ± s.e.m.). **c**, Absolute numbers of B cell precursors in the bone marrow (one femur and one tibia) (*n* = 6–12 mice per genotype, where each data point is a single mouse). Significance was determined by an unpaired two tailed *t*-test (mean ± s.e.m.). **d**, Absolute numbers of mature IgM^+^IgD^+^ B cells in the spleen (*n* = 4 mice per genotype, where each data point is a single mouse). Significance was determined by an unpaired two tailed *t*-test (mean ± s.e.m.). Source data

**Extended Data Fig. 4:**
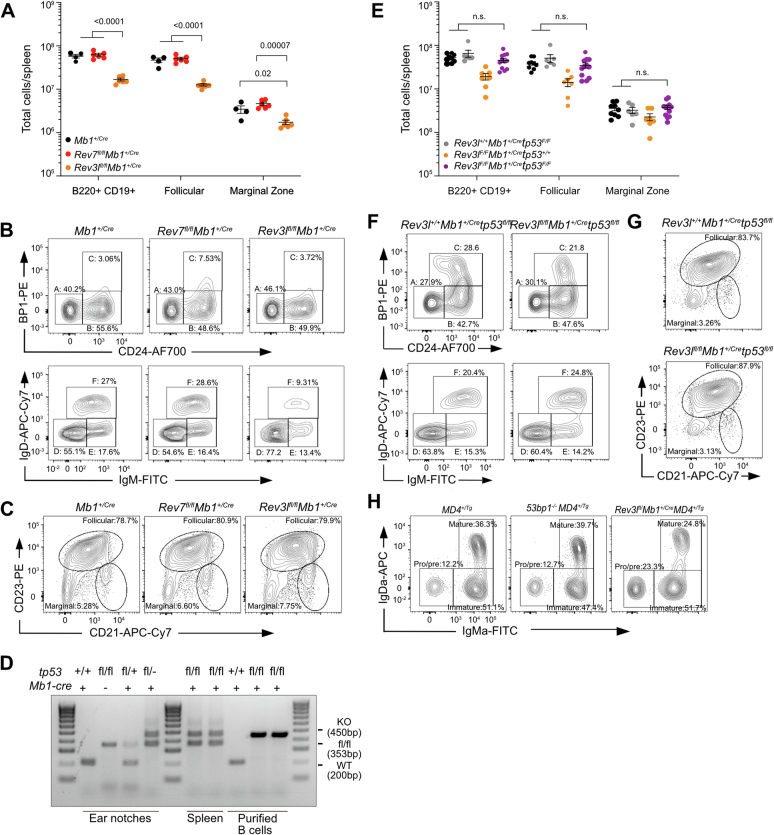
REV7-independent and NHEJ-independent functions of Pol ζ support B cell development. **a)** Flow cytometric sub-classification of nature splenic b cell fractions. n = 4-6 mice per genotype, where each data point is a single mouse. Significance was determined by unpaired two tailed *t*-test, Mean±SEM. **b)** Flow cytometry analysis of B cell development in the bone marrow of *Mb1*^*+/Cre*^, *Rev7*^*f/lf*^ *Mb1*^*+/Cre or*^ *Rev3l*^*fl/fl*^ *Mb1*^*+/Cre*^ *mice*; gating on B220^+^CD43^+^ (top, Hardy fractions A, B and C) and on B220^+^CD43^−^ (bottom, Hardy fractions D, E and F). Representative data; *n* = 3 experiments. **c)** Flow cytometry analysis of mature b cell populations in the spleen of *Mb1*^*+/Cre*^, *Rev7*^*f/lf*^ *Mb1*^*+/Cre or*^ *Rev3l*^*fl/fl*^ *Mb1*^*+/Cre*^ *mice*; gating on B220^+^CD19^+^. Representative data; *n* = 3 experiments. **d)** Representative image of PCR amplicons from genomic DNA obtained by ear biopsies (left), splenic b cells or purified splenic b cells (right) from mice of the indicated genotype. Bands of different size correspond to the *p53*^*fl*^ allele (353 bp), *p53*^*−/−*^ allele (450 bp) and *WT* allele (200 bp). n = >20 mice per genotype except for purified B cells; n = 2 independent experiments each with 2-3 mice. **e)** Flow cytometric sub-classification of nature splenic b cell fractions. n =6-12 mice per genotype, where each data point is a single mouse. Significance was determined by unpaired two tailed *t*-test, Mean±SEM. **f)** Flow cytometry analysis of B cell development in the bone marrow of *Rev*^*+/+*^ *p53*^*fl/fl*^ *Mb1*^*+/Cre or*^ *Rev3l*^*fl/fl*^ *p53*^*fl/fl*^ *Mb1*^*+/Cre*^ *mice*; gating on B220^+^CD43^+^ (top, Hardy fractions A, B and C) and on B220^+^CD43^*−*^ (bottom, Hardy fractions D, E and F). Representative data; *n* = 3 experiments. **g)** Flow cytometry analysis of mature b cell populations in the spleen of *Rev*^*+/+*^ *p53*^*fl/fl*^ *Mb1*^*+/Cre or*^ *Rev3l*^*fl/fl*^ *p53*^*fl/fl*^ *Mb1*^*+/Cre*^ *mice*; gating on B220^+^CD19^+^. Representative data; *n* = 3 experiments. **h)** Flow cytometry analysis of B cell development in the bone marrow of MD4^+/Tg^, Rev3^fl/fl^ Mb1^+/Cre^ MD4^+/Tg^, 53BP1^*−*/*−*^ MD4^+/TG^ mice; gating on B220^+^. Representative data; *n* = 3 experiments. Source data

Despite the proliferative capacities of *Rev3l*^*∆/∆*^ *Mb1*^+/Cre^ stimulated mature B splenocytes, the staging of B lineage cells in the bone marrow of *Rev3l*^*F/F*^ *Mb1*^+/Cre^ mice revealed developmental abnormalities. In these mice, diminished frequencies in pro B cell stage cells persisted throughout the small pre-B cell, immature B cell and mature circulating B cell stages and were noticeably more severe than those observed in *53bp1*^*−*/*−*^ mice (Fig. 4a and Extended Data Figs. 1h,n and 4a–c). This also contrasted with *Rev7*^*F/F*^ *Mb1*^+/Cre^ mice, where *Mb1-cre*-mediated deletion of *Rev7* in B cells caused no development phenotypes (Fig. 4a)^11^, confirming that Pol ζ’s function in developing B cells requires its Pol REV3L but not its accessory subunit REV7. To determine the cause of B cell attrition in *Revl3*^*F/F*^ *Mb1*^+/Cre^ mice, we generated *Rev3l*^*F/F*^ *p53*^*F/F*^ *Mb1*^+/Cre^ mice (Extended Data Fig. 4d). p53 deletion is known to suppress DNA damage-dependent apoptosis in murine lymphocytes^41^; likewise, its disruption rescued B cell frequencies in the bone marrow and spleen of *Rev3l*^*F/F*^ *p53*^*F/F*^ *Mb1*^+/Cre^ mice (Fig. 4b,c and Extended Data Fig. 4e–g). By contrast, the expression of a transgenic B cell receptor specific for hen egg lysosome (*MD4*^*+/Tg*^) did not rescue B cell frequencies in *Rev3l*^*F/F*^ *Mb1*^+/Cre^ mice but did so in a V(D)J recombination-defective *53bp1*^*−/−*^ background (Fig. 4d and Extended Data Fig. 4h). On this basis, we surmise that, in the *Rev3l-*deleted background B cell lineage, abnormalities are most likely precipitated by replication-associated DNA damage and not by defects in V(D)J recombination. Thus, Pol ζ supports B lymphocyte development in a REV7-independent and DNA end-joining-independent manner. The rescue of B cell development conferred by p53 deletion is consistent with Pol ζ’s known role in suppressing DNA replication-associated genomic stability^42^, providing new evidence of REV7-independent functions of Pol ζ in genome maintenance.

### Limited role for shieldin in *Brca1*-deficient mouse embryos

**Fig. 5:**
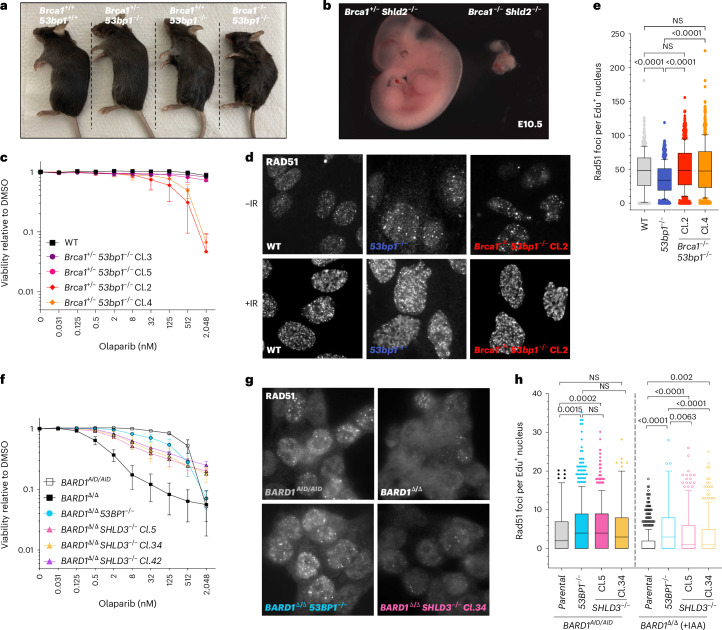
53BP1-dependent HR suppression in *BRCA1-*deficient cells is shieldin independent. **a**, Representative image of *53bp1*^*−/−*^*Brca1*^*−/−*^ mice and relevant controls at ~12 weeks. Genotypes are indicated. **b**, Representative image of *a Shld2*^*−/−*^*Brca1*^*+/−*^ control embryo and *Shld2*^*−/−*^*Brca1*^*−/−*^ double-knockout embryo at E10.5. **c**, Survival of the indicated MEF cell lines grown for 7 days in the presence of the indicated doses of olaparib (*n* = 3 biological experiments; mean ± s.d.). **d**, Immunofluorescence microscopy of Rad51 IRIF in indicated MEF cell lines. Cells were irradiated (5 Gy) and fixed 2 h later. Representative images of *n* = 3 biological experiments. **e**, Quantification of RAD51 IRIF in Edu^+^ cells from **d**. Integrated intensity and foci quantifications were made using CellProfiler. Boxes indicate the 25th–75th percentiles with the median denoted and whiskers indicate the 10th–90th percentiles. Significance was determined by a two-sided Kruskal–Wallis *H* test with Dunn’s correction for multiple comparisons. ****P < 0.0001 and **P < 0.005 (*n* = 3 biological experiments). **f**, Survival of the indicated *BARD1*^*AID/AID*^ HCT-116 cell lines grown for 7 days in the presence or absence of IAA (1 mM), doxycycline (2 mg ml^*−*1^) and the indicated doses of olaparib (*n* = 3 biological experiments; mean ± s.d.). **g**, Immunofluorescence microscopy of Rad51 IRIF in indicated *BARD1*^*AID/AID*^ cell lines. Cultures were supplemented with doxycycline (2 mg ml^*−*1^ for 24 h) before the addition of IAA (1 mM). After 24 h, cells were irradiated (5 Gy) and fixed 2 h later. Representative images of *n* = 3 biological experiments. **h**, Quantification of RAD51 IRIF in Edu^+^ cells from **g**. Integrated intensity and foci quantifications were made using CellProfiler. Boxes indicate the 25th–75th percentiles with the median denoted and whiskers calculated by the Tukey method. Significance was determined by a two-sided Kruskal–Wallis *H* test with Dunn’s correction for multiple comparisons (*n* = 4 biological experiments). Source data

The above findings confirm that the 53BP1 pathway directs multiple mechanistically distinct activities, which can be distinguished by shieldin–CST involvement. Synergy between these activities is required for the efficient joining of AID-induced DSBs during CSR; however, during V(D)J recombination, 53BP1 acts independently of shieldin, CST or REV3L. We reasoned that these activities might, therefore, each contribute separately to the pathological events in *Brca1-*deficient mice and tumors, where the 53BP1 pathway impedes HR and, through shieldin and CST, mediates toxic NHEJ events^11,16,21,22,24,26,27,29^. To test this, we interbred *53bp1* and *Shld2* double-knockout mice with mice harboring *Brca1* exon 5–13 deletions (*Brca1*^*+/−*^) that ablate all BRCA1 protein expression^43^. *Brca1*^*−/−*^ mouse embryos die early in development, with none outlasting embryonic day 7.5 (E7.5)^44^. However, embryonic lethality can be circumvented upon deletion of *53bp1*, yielding viable, albeit tumor-prone mice^19,20^. In our hands, *Brca1*^*−/−*^*53bp1*^*−/−*^ mice were born at around half of the expected frequency (Fig. 5a and Table 2) and weighed less than their *Brca1*^*+/−*^*53bp1*^*−*/*−*^ littermates, with most succumbing to thymic lymphoma between 12 and 20 weeks of age, consistent with recent reports^20,45^. By contrast, no *Brca1*^*−/−*^*Shld2*^*−/−*^ double-knockout pups were born to intercrosses between *Brca1*^*+/−*^*Shld2*^*−/−*^ mice (Table 2). Furthermore, we recovered only non-viable *Brca1*^*−/−*^*Shld2*^*−*/*−*^ embryos between stages E8.5-E11.5, all of which exhibited severe developmental delay (Fig. 5b and Table 2). Of note, from staged embryos obtained in *Brca1* heterozygous intercrosses performed in parallel (analyzed at E8.5 and E10.5), no *Brca1*^*−/−*^ embryos were recovered. This implies that, unlike the case for *53bp1* deficiency, *shieldin* inactivation cannot restore viability in *Brca1*-null mice and only modestly delays the onset of embryonic lethality.

**Table 2:** *Brca1*^*−/−*^ intercrosses

*Brca1*^*+/−*^*53bp1*^*−/−*^ × *Brca1*^*+/−*^*53bp1*^*−/−*^			
*Brca1*	+/+	+/−	−/−
*53bp1*	+/+	−/−	−/−
Live pups (*n* = 209)	68 (52.25)	120 (104.5)	21 (52.25)
***Brca1***^***+/−***^***Shld2***^−/−^ **×** ***Brca1***^***+/−***^***Shld2***^−/−^			
*Brca1*	+/+	+/−	−/−
*Shld2*	+/+	^−/−^	^−/−^
Live pups (*n* = 242)	89 (60.5)	153 (121)	0 (60.5)
Embryos, (E8.5–11.5) (*n* = 57)	15 (14.25)	32 (28.5)	10 (14.25)

Summary of the frequency of live pups born or >E12.5 embryos genotyped from *53bp1*^−/−^*Brca1*^+/−^ or *Shld2*^−/−^*Brca1*^+/−^ crosses.

### DSB repair pathway choice control is shieldin independent

**Extended Data Fig. 5:**
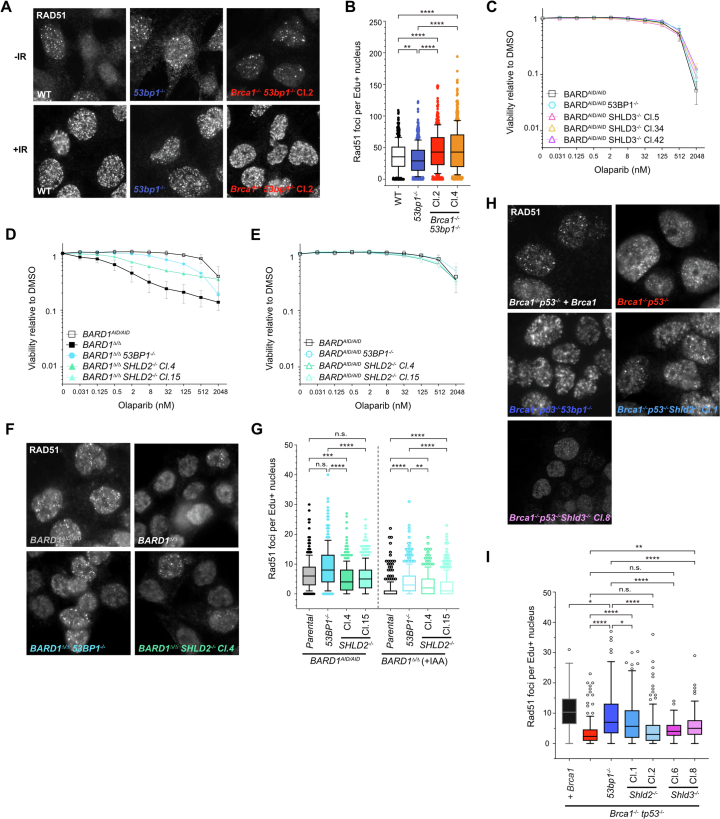
53BP1-dependent HR suppression in BRCA1-deficient cells is shieldin independent. **a)** Immunofluorescent microscopy of Rad51 IRIF in indicated MEF cell lines using Rabbit serum gifted by R. Kanaar. Cells were irradiated (5 Gy) and fixed 2 h later. Representative images of n = 3 biological experiments. **b)** Quantification of RAD51 IRIF in Edu^+^ cells from D. Integrated intensity and foci quantifications were made using CellProfiler. Boxes indicate the 25th–75th percentiles with the median denoted, and whiskers indicate the 10^th^-90^th^ percentile. Significance was determined by two-sided Kruskal–Wallis H test with Dunn’s correction for multiple comparisons. p values; ****p < 0.0001, **p < 0.0005, **p < 0.005, *p < 0.05. n = 3 biological experiments. **c)** Survival of the indicated BARD^AID/AID^ cell lines grown for 7 days in the presence of doxycycline (2 μg ml^*−*1^) and the indicated doses of olaparib, n = 3 biological experiments, Mean±SEM. **d)** Survival of the indicated *BARD1*^*AID/AID*^ HCT-116 cell-lines grown for 7 days in the presence or absence of IAA (1mM), doxycycline (2 mg ml^*−*1^) and the indicated doses of Olaparib, *n* = *3* biological experiments, Mean±SD. **e)** Survival of the indicated *BARD1*^*AID/AID*^ HCT-116 cell-lines grown for 7 days in the presence or absence of IAA (1mM), doxycycline (2 mg ml^*−*1^) and the indicated doses of Olaparib, *n* = *3* biological experiments, Mean±SD. **f)** Immunofluorescent microscopy of Rad51 IRIF in indicated BARD1^AID/AID^ cell lines. Cultures were supplemented with doxycycline (2 mg ml^*−*1^ for 24 h) before addition of IAA (1 mM). After 24 h cells were irradiated (5 Gy) and fixed 2 h later. Representative images of n = 3 biological experiments. **g)** Quantification of RAD51 IRIF in Edu^+^ cells from F. Integrated intensity and foci quantifications were made using CellProfiler. Boxes indicate the 25th–75th percentiles with the median denoted, and whiskers calculated by the Turkey method. Significance was determined by two-sided Kruskal–Wallis H test with Dunn’s correction for multiple comparisons. p values; ****p < 0.0001, ***p < 0.0005, **p < 0.005, *p < 0.05. **h)** Immunofluorescent microscopy of Rad51 IRIF in indicated KB1P-G3 mouse mammary tumour cell lines. Cells were irradiated (5 Gy) and fixed 2 h later. Representative images of n = 3 biological experiments. **i)** Quantification of RAD51 IRIF in Edu^+^ cells from H. Integrated intensity and foci quantifications were made using CellProfiler. Boxes indicate the 25th–75th percentiles with the median denoted, and whiskers calculated by the Turkey method. Significance was determined by two-sided Kruskal–Wallis H test with Dunn’s correction for multiple comparisons. p values; ****p < 0.0001, ***p < 0.0005, **p < 0.005, *p < 0.05. Source data

As shieldin loss blocked CSR but could not rescue the development of *Brca1-*null embryos, we hypothesized that the pro-NHEJ and HR-suppressive activities of the 53BP1 pathway might be distinguished at the level of shieldin involvement. Suggestive of 53BP1 operating independently of shieldin during HR suppression, we were unable to derive viable embryonic fibroblast (MEF) or embryonic stem cell (mESC) cell lines from *Brca1*^*−/−*^*Shld2*^*−*/*−*^ E8.5 embryos and blastocyst outgrowths, respectively. We, therefore, resorted to addressing shieldin’s involvement in engineered *BARD1*^*AID/AID*^ HCT-116 cells where BRCA1 can be conditionally inactivated through auxin degron-mediated degradation of its obligate binding partner protein BARD1 (refs. ^14,15^). Direct binding of DSB-proximal nucleosomes ubiquitinated on H2A Lys15 (H2AK15ub) by BARD1 is a prerequisite for BRCA1-dependent pathway choice control because it directly inhibits 53BP1–chromatin interactions at DNA damage sites^14,17^. Accordingly, in auxin-treated *BARD1*^*AID/AID*^ HCT-116 cells (hereforth referred to as *BARD1*^Δ/Δ^ cells), HR is fully restored upon deletion of 53BP1, resulting in a dramatic (albeit incomplete) suppression of cellular hypersensitivity to PARPi olaparib^14^. Mirroring the case in *BARD1*^*∆/∆*^ HCT-116, mouse embryonic fibroblast cell lines derived from *Brca1*^*−/−*^*53bp1*^*−/−*^ embryos exhibited similar levels of residual olaparib sensitivity (Fig. 5c) yet WT levels of RAD51 recruitment upon irradiation (as visualized with two different RAD51 antibodies; Fig. 5d,e and Extended Data Fig. 5a,b), again indicative of HR restoration. These consistent results in murine and human cell lines reassured us of the suitability of *BARD1*^*AID/AID*^ HCT-116 as a cellular model to investigate and compare the contributions of 53BP1 and shieldin to *BRCA1* deficiency-associated HR defects.

In line with previous results in *BRCA1-*deficient cells^11,22,26^, deletion of *SHLD2* or *SHLD3* in *BARD1*^Δ/Δ^ cells markedly enhanced cell survival in olaparib cytotoxicity assays. Importantly, however, olaparib resistance was less penetrant in *BARD1*^Δ/Δ^*SHLD3*^*−/−*^ and *BARD1*^Δ/Δ^*SHLD2*^*−/−*^ cells, as evidenced by the enhanced relative survival of *BARD1*^Δ/Δ^*53BP1*^*−/−*^ cells in these experiments (Fig. 5f and Extended Data Fig. 5c–e). HR defects in replicating (S phase staged) *BARD1*^Δ/Δ^*SHLD3*^*−/−*^ cells and *BARD1*^Δ/Δ^*SHLD2*^*−/−*^ cells were also evident at the level of defective RAD51 recruitment into ionizing radiation-induced foci (IRIF), in stark contrast to *BARD1*^*Δ/Δ*^*53BP1*^*−/−*^ cells, where RAD51 IRIF accumulated at WT (non-auxin-treated *BARD1*^*AID/AID*^ cells) levels (Fig. 5g,h and Extended Data Fig. 5f,g). Furthermore, consistent differences in RAD51 recruitment were observed in irradiated KB1P-G3-derived *Brca1*^*−/−*^*trp53*^*−/−*^ murine mammary tumor cell lines polyclonally deleted for either *53bp1* (ref. ^46^) or shieldin genes *Shld2* (ref. ^22^) or *Shld3* (ref. ^11^) (Extended Data Fig. 5h,i).

**Fig. 6:**
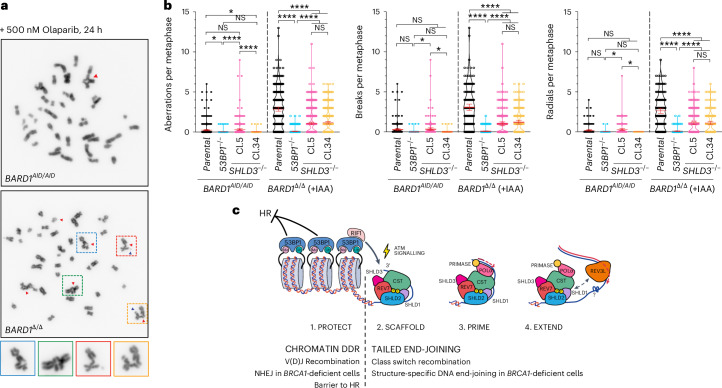
Independent and synergistic contributions of 53BP1 and shieldin to genomic instability in *BRCA1-*deficient cells. **a**, Representative metaphase images from the indicated *BARD1*^*AID/AID*^ cell lines. Cultures were supplemented with doxycycline (2 mg ml^*−*1^for 24 h) before the addition of IAA (1 mM). After a further 24 h, cells were treated with 500 nM olaparib and fixed 24 h later. Representative images of *n* = 3 biological experiments. **b**, Quantification of 40–65 metaphases per genotype per experiment. Significance was determined by a two-sided Kruskal–Wallis *H* test with Dunn’s correction for multiple comparisons. Horizontal bars indicate the mean values ± 95% confidence interval. ****P < 0.0001, ***P < 0.0005, **P < 0.005 and *P < 0.05 (*n* = 3 biological experiments). **c**, Model for 53BP1-dependent DSB repair. Chromatin-associated 53BP1 complexes suppress HR and promote V(D)J recombination. DNA damage-dependent signaling by RIF1 is conferred to shieldin through interactions with SHLD3. DSB signaling by ATM stimulates the assembly of shieldin–CST complexes on ssDNA, promoting the recruitment of Pol α–primase to initiate fill-in synthesis. Pol ζ REV3L increases the processivity of Pol α–primase-catalyzed fill-in synthesis. Source data

**Extended Data Fig. 6:**
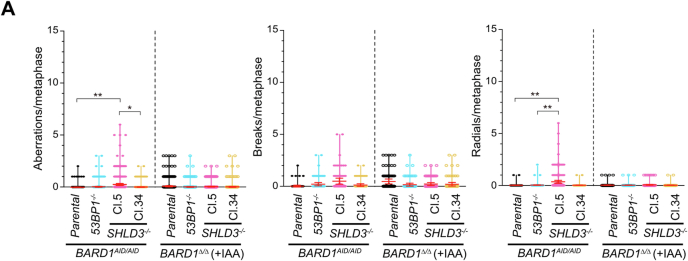
Independent and synergistic contributions of 53BP1 and shieldin to genomic instability in BRCA1-deficient cells. **a)** Quantification of between 40 and 65 metaphases analysed per genotype/experiment. Cultures were supplemented with doxycycline (2 μg ml^*−*1^ for 24 h) before addition of IAA (1 mM). After a further 24 h cells were treated with DMSO and fixed 24 h later. Significance was determined by two-sided Kruskal–Wallis *H* test with Dunn’s correction for multiple comparisons. p values; ****p < 0.0001, ***p < 0.0005, **p < 0.005, *p < 0.05. All comparisons not indicated are not significant. n = 3 biological experiments. Source data

The different extents to which inactivation of 53BP1 or shieldin could suppress DNA repair defects in *BRCA1*-deficient *BARD1*^Δ/Δ^ cells was also evident at the level of chromosome stability. In metaphase chromosome spreads from *BARD1*^Δ/Δ^ cells, olaparib treatment induced high frequencies of chromosome or chromatid breaks and radial chromosomes (Fig. 6a,b and Extended Data Fig. 6). Chromosome breaks and radials were only slightly reduced in frequency in *BARD1*^Δ/Δ^*SHLD3*^*−*/*−*^ cells, highlighting 53BP1’s retained capacity to block repair and mediate toxic joining events independently of shieldin. By contrast, both classes of chromosome lesion were nearly completely suppressed in *BARD1*^Δ/Δ^*53BP1*^*−*/*−*^ cells (Fig. 6a,b), consistent with their restored capacity for HR (Fig. 5f,g and Extended Data Fig. 5f,g). We can, therefore, conclude that HR inactivation in *BRCA1*–*BARD1-*deficient cells is more profoundly dictated by 53BP1 status than the status of shieldin or its cooperating proteins. Nevertheless, our results show that, in the absence of BRCA1-dependent HR promotion, shieldin-directed DNA end joining exacerbates DNA damage-induced chromosomal instability, where it also makes substantial contributions to the overall hypersensitivity of cancer cells to HR deficiency-targeting PARPis (Fig. 6c).

## Discussion

In this study, we adopted a genetic approach to systematically dissect the 53BP1 pathway. By comparing the traits of mice deficient in 53BP1, shieldin or CST, we aimed to elucidate the intricate relationship between 53BP1 and its downstream DNA-processing components in both normal and pathological contexts. Our findings shed light on the remarkable capability of 53BP1 to facilitate DSB repair even in the absence of shieldin. However, they also establish the indispensability of shieldin–CST interplay for efficient 53BP1-dependent DNA end joining during CSR and, furthermore, confirm Pol ζ’s involvement in these important DNA synthesis-dependent DNA end-joining reactions.

### Shieldin is a DSB repair-specific mediator of CST

Upon binding ssDNA at telomeres and during DNA repair, CST acts as a catalyst of Pol α–primase-dependent DNA synthesis^29,47^. The discovery of shieldin–CST interdependence during CSR, thus, underscores the specialized nature of fill-in DNA synthesis as a prerequisite for the joining of AID-dependent DSBs. We additionally demonstrate that 53BP1’s reliance on shieldin–CST differs dramatically across different repair contexts. We posit that these differences reflect the unique DNA end-processing demands inherent to the spectrum of DSB structures generated in each distinct scenario, coupled with their varying frequencies of occurrence.

These conclusions are supported by multiple parallel observations in lymphocytes, mice and cancer cell models. In addition to the equivalent severity of class-switching defects in mature B cells from *Shld2*^*−/−*^, *Shld3*^*−/−*^ and *Ctc1*^*F/F*^ *Mb1*^*+/cre*^ mice, hypothesis-agnostic elucidation of the shieldin interactome in B cells identified CST proteins as its primary interaction partners. Interestingly, interactions between shieldin and CST are induced upon DNA damage, a behavior explained by its reliance on the DSB-responsive kinase ATM. Upstream of shieldin, ATM-dependent phosphorylation of consensus Ser-Glu-containing motifs in 53BP1 promote the recruitment of RIF1 (refs. ^7,48^) by a recently discovered phospho-ligand-binding module in the RIF1 heat repeats^8^. Given the absence of additional shieldin interactors in our proteomics datasets, the ATM-dependent phosphorylation events that guide shieldin-dependent CST recruitment to DNA damage sites most likely involve a cryptic phospho-ligand-binding module within either shieldin or CST. From a structural perspective, it is noteworthy that neither CSR nor the ATM-dependent shieldin–CST interaction discovered here relies on a recently described N-terminal CTC1-binding motif in SHLD1 (ref. ^29^). Indeed, we found that a SHLD1^∆exon1^ protein deleted of its first 59 amino acids (encompassing this motif at aa 18–21) efficiently retrieved CST from lysates when they were prepared from irradiated B cells (Extended Data Fig. 2f). Similarly, mature splenic B cells from *Shld1*^*∆exon1*^ mice (a gift from the MMRRC^49^) confirmed to express an analogous truncated protein were also found to support near-WT class-switching frequencies in ex vivo stimulation experiments (Extended Data Fig. 2g,h), consistent with our previous results in *Shld1*^*−/−*^ CH12-F3 cells expressing a *Shld1*^*∆N*^ transgene^29^. Nevertheless, ATM-dependent shieldin–CST interactions are similarly consistent with shieldin’s importance in recruiting CST to DSB termini, explaining their interdependence in downstream DNA end joining.

Interestingly, this concept draws intriguing parallels with the regulation of CST-dependent C-strand fill-in synthesis at telomeres, governed by the shelterin complex^50^. In the telomeric context, the recruitment of CST to telomeres hinges on direct interactions between CST and the shelterin protein protection of telomere 1b (POT1b)^47^. Recent structural findings revealed that this involves a multisite interaction between CTC1 and regions within the triple OB-fold domain in POT1, which is further potentiated by POT1 phosphorylation^51^. A similar triple OB-fold architecture is also present in SHLD2 (refs. ^11,21,22,26^); thus, it is tempting to speculate that a similar mode of binding between CTC1 and structurally related features in POT1 or SHLD2 might underpin the initiation of fill-in DNA synthesis at telomeres and DSBs, respectively^27,29,47^.

### Pol ζ acts downstream of shieldin–CST during DNA end joining

Using mice codeleted for *Rev3l* and either *Shld2* or *Rev7*, we additionally detected an epistatic relationship between these genes in class-switching mature B cells. This offers a rational mechanistic explanation for Pol ζ’s role in NHEJ^39^, positioning it downstream of shieldin–CST, most likely in the extension of DNA repair synthesis that follows priming by Pol α–primase. Class switching in *Rev3l-*deficient B cells occurs at ~50% of WT frequencies, a level ~3–4-fold higher than that seen in those lacking *shieldin* genes or *Ctc1*. Does this indicate that Pol ζ is not the sole DNA Pol acting downstream of CST–Pol α–primase? As the typical extender of lagging-strand primers during DNA replication, Pol δ would be a likely redundant Pol. It is also possible that RNA–DNA primer synthesis on a short 5′ resected DNA end might be sufficient to stimulate the ligation of ssDNA-tailed DSBs by NHEJ, a pathway capable of ligating DSBs with ribonucleotide-incorporated termini^52^. Nevertheless, AID’s action during CSR generates ssDNA-tailed DSBs within switch (*S*) regions in the *Igh* locus, G+C-rich repeat-dense intronic elements abundant in palindromic motifs. The propensity of *S*-region ssDNA to form stem loops and/or parallel four-stranded G-quartets is well documented and such properties are speculated to contribute to various aspects of *S-*region function during CSR^53^. The formation of secondary DNA structures in the ssDNA-tailed termini of AID-dependent DSBs might similarly impede DNA synthesis by the canonical lagging-strand Pol δ, warranting the utility of a versatile TLS Pol to mediate DNA fill-in synthesis at *Igh* DSBs. Pol ζ also limits DNA damage accumulation during normal replication^42^, a function that we suggest extends to developing B cells that surprisingly does not involve REV7. Whether Pol ζ participates in other CST–Pol α–primase-catalyzed fill-in reactions remains to be explored. Such an interplay might be advantageous during C-strand fill-in at telomeres, where secondary DNA structures on the template G-strand could pose similar challenges to Pol δ processivity^54^.

### 53BP1 promotes DNA end joining independently of shieldin–CST

On the basis of the genetic analyses described above, we propose a refined model for the shieldin-dependent branch of the 53BP1 pathway (Fig. 6c). While vital for optimal CSR, the residual switching capacity of stimulated shieldin–CST-deficient B splenocytes notably exceeds that in *53bp1*-deficient B cells, confirming that 53BP1 promotes the repair of a small subset of AID breaks without the requirement for fill-in synthesis. An additional paradigm for shieldin-independent NHEJ is V(D)J recombination, where 53BP1 supports the development of normal frequencies of B and T lymphocytes by enhancing long-range joining events^10,11,13^. Why does 53BP1 operate independently of shieldin–CST during V(D)J recombination? The simple explanation for this is RAG-induced DNA breaks have no utility for shieldin–CST-dependent fill-in synthesis yet profit from a distinct activity exerted by 53BP1. Our comparative investigations of 53BP1 and shieldin functions in *BRCA1*-deficient cells provide additional evidence in support of this proposition. Shieldin inactivation confers a degree of PARPi resistance to *BARD1-*depleted cells, yet resistance was further increased by deletion of 53BP1. Similar distinctions were perhaps hinted at by murine tumor studies. The potent survival benefit conferred by PARPi treatments in mice implanted with *Brca1*^*−/−*^*Trp53*^*−/−*^ mammary tumors was completely lost when tumours were additionally deleted of *53bp1* (ref. ^55^); however, they modestly extended lifespan in equivalent tumor engraftment experiments when the tumors were instead deleted for *Shld1* or *Shld2* (ref. ^22^). Our knockout experiments in *BARD1*^*∆/∆*^ cells also revealed 53BP1 as the predominant mediator of PARPi-induced chromosome breaks and radial chromosomes, with shieldin making a lesser, albeit substantial and important contribution, in agreement with previous findings^11,21,22,26–29^. Given the different contributions that 53BP1 and shieldin make to the repair of RAG-induced and AID-induced DSBs in lymphocytes, we speculate that DSBs resulting from PARPi-induced replication blocks might likewise comprise a spectrum of DNA end structures, of which only a subset necessitate shieldin–CST axis-dependent repair.

### HR suppression is a shieldin-independent function of 53BP1

One of the most striking deviations in phenotypes conferred by 53BP1 and shieldin losses was at the level of HR modulation in *BRCA1*–*BARD1-*deficient human and mouse cell lines. Here, 53BP1 deletion fully restored RAD51 recruitment to WT levels, indicative of fully competent HR. By contrast, the profound RAD51 recruitment defects that characterized human *BARD1*^*∆/∆*^ HCT-116 cells and murine KB1P tumor cells were only slightly ameliorated in *shieldin-*inactivated derivative cell lines. This distinction implies that, while DSB repair pathway choice regulation is dictated by 53BP1, it is only modestly influenced by downstream shieldin–CST-mediated DNA end processing. The weak degree of observed phenotypic suppression in *Shld2*^*−/−*^*Brca1*^*−/−*^ embryos offers the most compelling support for this conclusion. Shieldin disruption only slightly delayed the onset of embryonic lethality, in stark contrast to the viability and superficially normal postnatal development of *53bp1*^*−/−*^*Brca1*^*−/−*^ mice. These findings may seem in contradiction to a recent report, where *Shld2* knockout rescued embryonic and postnatal development in mice homozygous for the hypomorphic *Brca1*^*∆11*^ mutation^12^. However, these differences are almost certainly attributable to the notable residual functionality of the BRCA1^∆11^ protein. BRCA1^∆11^–BARD1 complexes are readily recruited to sites of DNA damage and retains BRCA1 functional domains including its coiled coil that links it to PALB2 (partner and localizer of BRCA2)–BRCA2 (ref. ^56^). However, DNA end resection is severely reduced in cells deleted of exon 11 in *BRCA1* (ref. ^56^), a defect that might interfere with HR when challenged with antagonistic 53BP1-directed shieldin–CST fill-in activities.

In summary, our study reveals a surprising segregation of duties between 53BP1 and its downstream effector proteins during DSB repair. Shieldin–CST-dependent fill-in synthesis at DNA ends constitutes an ancillary reaction that supports the joining of DSBs with recessed ssDNA termini, such as those generated following the processing of staggered DNA cleavages induced by AID during CSR. Outside of CSR, however, 53BP1-dependent DSB repair remains remarkably resilient to loss of these effectors, consistent with a direct stimulation of DNA repair by chromatin-bound 53BP1 complexes. Our results also implicate upstream 53BP1 complexes, but not their DNA-processing effectors, as the major determinants of HR suppression in mammalian cells (Fig. 6c). This concept aligns well with our recently proposed chromatin-centric model for DSB pathway choice control, where HR and NHEJ outcomes are dictated by a mutual antagonism between BRCA1–BARD1 and 53BP1, upon their engagement of related DSB-proximal chromatin signatures^14,15^. Future work will be needed to address whether this hierarchy in the 53BP1 pathway will also be reflected in the clinical penetrance of mutations affecting *53BP1* and its effector genes in therapy-resistant *BRCA1-*mutant cancers.

## Methods

### Mice

The production and breeding of the genetically altered mice was carried out in accordance with the UK Home Office Animal (Scientific Procedures) Act 1986, with procedures reviewed by the Clinical Medicine Animal Welfare and Ethical Review Body at the University of Oxford, and conducted under project license PP8064604. Animals were housed in individually ventilated cages, provided with food and water ad libitum and maintained on a 12-h light–dark cycle (150–200 lx). The only reported positives on Federation of European Laboratory Animal Science Associations health screening over the entire time course of these studies were for *Helicobacter hepaticus and Entamoeba* spp. Experimental groups were determined by genotype and were, therefore, not randomized, with no animals excluded from the analysis. Sample sizes for fertility studies were selected on the basis of previously published studies and all phenotypic characterization was performed blind to the experimental group. All mice used in this study were generated on or backcrossed onto a C57BL/6 background (>5 generations). F_1_ mice harboring the *Shld2*^*tm1a*^ (*Shld2*^*tm1a(EUCOMM)Hmgu*^, MGI: 5428631) allele were generated by backcrossing chimeric F_0_ mice generated by microinjection of gene-targeted C57BL/6N (JM8F6) derived mESC cells into albino C57BL6/J blastocysts. The targeting was performed in house using the targeting vector from the European Conditional Mouse Mutagenesis Program for the International Mouse Knockout Consortium, following the consortium’s recommended screening conditions. The resulting *Shld2*^*tm1a*^ mice were then crossed with Flp-Cre-positive mice (Tg(*ACTB*-*Flpe*)9205Dym; Jax stock 005703) to generate a conditional allele with LoxP sites flanking exons 4 and 5, which includes the translation start site^35^. A targeted *Shld2*-null allele was subsequently generated by germline recombination of the LoxP sites using PGK-cre^57^ (B6.C-Tg(Pgk1-cre)1Lni/CrsJ; Jax stock 020811) (Fig. 1a). *Shld3*-null mice were generated at MRC Harwell by microinjection of Cas9 ribonucleoprotein complexes targeting exon 2 in the C57BL/6 background. Two founder mice, *Shld3*^*DEL2061−EM1*^ and *Shld3*^*DEL2061-EM2*^, with deletions of the sole coding exon, were generated (2,054 bp and 2,061 bp, respectively) (Fig. 1a). Helmholtz Munich/German Research Center for Environmental Health provided the *Ctc1*^*tm1a(KOMP)Wtsi*^ mutant mouse line (allele: C57BL/6N-Ctc1<tm1a(KOMP)Wtsi>/Ieg). INFRAFRONTIER/EMMA (www.infrafrontier.eu (ref. ^58^)) and Helmholtz Zentrum Munich/German Research Center for Environmental Health distributed the sperm that was used to rederive the mouse line (EM: 10068) in our host institution’s transgenics core facility (MRC WIMM). Conditional *Ctc1*^*F*/*F*^ mice were generated by crossing the ‘knockout-first’ allele (*Ctc1*^*tm1a(KOMP)Wtsi/Ieg*^*;* MGI: 4363331) to Flp-Cre-positive mice (Tg(*ACTB*-*Flpe*)9205Dym; Jax stock 005703) to generate mice with the *Ctc1*^*tm1c*^ conditional allele. Experimental *Ctc1*^*F/F*^ *Mb1*^+/Cre^, *p53*^*F/F*^ *Mb1*^+/Cre^ (Trp53^floxed^; MGI: 98834) and *Rev3L*^*F/F*^ *Mb1*^+/Cre^ (Rev3l^tm1Rsky^; MGI: 1337131) mice were generated by intercrossing with mice expressing the B cell lineage *Mb1*-cre deleter strain (*Cd79a*^*tm1(cre)Reth*^; MGI:368745)^40^. The *53bp1*^−/−^ mice (*Trp53bp1*^*tm1Jc*^; MGI: 2654201) and *Brca1*^*−/−*^ mice (Brca1^null^; MGI:104537) were generated as described elsewhere^19,43,59^. *Shld1*^*∆exon1*^ mice (*C57BL/6NCrl-1110034G24Rik*^*em1(IMPC)Mbp/Mmucd*^*;* stock 043862-UCD), obtained from the Mouse Biology Program (University of California, Davis) harbored a clustered regularly interspaced short palindromic repeats (CRISPR)–Cas9-generated deletion of the terminal 71 bp of *Shld1* exon 1. Complementary DNA sequencing confirmed the expression of a functional spliced *Shld1*^*∆exon1*^ transcript, initiated at an indel-generated in-frame ATG codon within the preserved frame-shifted sequences in exon 1 (ATG encoded within Tyr29–Glu30 codons).

All B cell phenotyping experiments involved age-matched 8–16-week-old male or female animals on an inbred C57BL/6 background. We did not pursue lower-penetrance phenotypes; thus, statistically significant data could typically be obtained with small group sizes (typically 4–10 mice). Randomization of samples was only undertaken during the scoring of chromosomal aberrations during metaphase analyses. Mice of a certain genotype were selected on the basis of a unique mouse identifier that does not indicate mouse genotype; thus, phenotype–genotype relationships were determined only at the data analysis stage. All experiments were approved by the University of Oxford Ethical Review Committee and performed under a UK Home Office Licence in compliance with animal use guidelines and ethical regulations.

### Immunizations

WT, *53bp1*^*−*/*−*^, *Shld2*^*−*/*−*^, *Shld3*^*−*/*−*^ and *Ctc1*^*F*/F^ *Mb1*^+/cre^ mice were immunized intraperitoneally with 50 mg of NP-CGG (Santa Cruz Biotechnologies) emulsified in Imject Alum adjuvant (Pierce, Thermo Fisher). Blood samples were collected from the tail vein at 0, 7, 14, 21 and 28 days after immunization.

### ELISA

ELISAs were used to quantify the production of NP-specific antibodies in mice serum. First, 96-well plates were coated with 1 μg ml^−1^ NP-BSA (Biosearch Technologies) in bicarbonate buffer, blocked with 5% milk in PBS and incubated with serial dilutions of serum collected at different time points from immunized mice. Plates were probed using alkaline phosphatase-coupled antibodies against mouse IgM and IgG1 (Southern Biotech). Phosphatase substrate (Sigma) was used for detection and optical density was measured at 405 nm. For IgG1, pooled blood from postimmunization WT mice was used as a standard and serially diluted into a standard curve. The first dilution was established as 1,000 arbitrary units (AU). For IgM, pooled blood from day 7 was used as a standard. Immunoglobulin concentrations in mouse serum or culture supernatants were determined by sandwich ELISA. Total IgG, IgM and IgA was measured with mouse IgG, IgM and IgA ELISA kits, respectively (Bethyl Laboratories), according to the manufacturer’s instructions. Mouse serum with known immunoglobulin concentrations of each immunoglobulin was used as a standard.

### Micronuclei analysis

Micronuclei from peripheral blood samples were analyzed as described by Balmus et al.^32^. Approximately 50 μl of tail-vein blood was collected into heparin solution and immediately fixed in cold methanol. Samples were stored at −80 °C until processing. Cells were then stained with CD71 (1:400; 113805, BioLegend) and 1 μl (1 mg ml^−1^) propidium iodide.

### Lymphocyte analysis and flow cytometry

Cell suspensions from bone marrow (one femur and one tibia), spleen and thymus were counted on a Pentra ES60 Hematology Analyzer (Horiba) and stained with anti-mouse antibodies against the following antigens as appropriate (all from BioLegend unless otherwise stated) in fluorescence-activated cell sorting (FACS) buffer (PBS with 2% BSA and 0.025% sodium azide): IgD (1:500; 405716 or Thermo Fisher 12-5993-82; 11-26c.2a), IgG1 (1:200; 406606, RMG1-1), IgM (1:500; 406506 or 406514, RMM-1), B220 (1:500; 103232, 103244, 103206 or 103212, RA3-6B2), BP1 (1:200; 108308, 6C3), CD4 (1:500; 100430), CD8a (1:500; 100732), CD19 (1:500; 115534 or 115520, 6D5), CD24 (1:1,000; 101827 or BD Pharmingen 562563, M1/69), CD93 (1:200; 136510, AA4.1), CD23 (1:200; BD Pharmingen 553139, B3B4), CD25 (1:500, 102008), CD43 (1:200; BD Pharmingen 562865, S7 or Thermo Fisher 11-0431-85, eBioR2/60), CD44 (1:200; 103022) and CD21 (1:500; BD Biosciences 563176, 7G6). Mouse BD F_c_ block (1:500, BD Pharmingen 553141) was added to block nonspecific binding and live–dead cells were discriminated after staining with Zombie Aqua viability dye (1:200; 423102). Data were acquired on an FACSCanto (BD Biosciences), LSR Fortessa X20 (BD Biosciences) or Attune NxT flow cytometer (Thermo Fisher) and were analyzed with FlowJo software version 10 (Tree Star). Gating strategies for bone marrow Hardy fractions are shown in Supplementary Fig. 1 and Extended Data Fig. 4.

### Ex vivo B splenocyte culture, stimulation and flow cytometry

B cells were purified from red blood cell-lysed single-cell suspensions of mouse spleens by magnetic negative selection using a B cell isolation kit (Miltenyi Biotec, 130-090-862). B cells (3 × 10^5^ per well in a 12-well plate) were cultured in RPMI supplemented with 10% fetal calf serum (FCS), 100 U per ml penicillin, 100 ng ml^−1^ streptomycin, 2 mM l-glutamine, 1× MEM nonessential amino acids, 1 mM sodium pyruvate and 50 μM β-mercaptoethanol. B cells were stimulated with 5 μg ml^−1^ lipopolysaccharide (LPS; Sigma, L7770-1MG), 10 ng ml^−1^ mouse recombinant interleukin (IL)-4 (Peprotech, 214-14-20) and agonist anti-CD40 antibody (0.5 μg ml^−1^; Miltenyl Biotec, FGK45.5). Cultures were grown at 37 °C with 5% CO_2_ under ambient oxygen conditions. Then, 4 days after seeding, stimulated B cells were analyzed using an FACSCanto or LSR Fortessa X20; analysis was performed using FlowJo. Cells were resuspended in FACS buffer, blocked with Mouse BD F_c_ block and immunostained with biotinylated antibodies as follows: anti-mouse IgG1 (1:100; BD Pharmingen 553441, A85-1), anti-mouse IgG2b (1:100; BioLegend 406704, RMG2b-1), anti-mouse IgG3 (1:100; BD Pharmingen 553401, R40-82) and streptavidin APC (1:500; Thermo Fisher 17-4317-82). Cells expressing IgE were assessed using anti-mouse IgE PE (1:200; BioLegend, 406908, RME-1). Live–dead cells were discriminated after staining with Zombie Aqua viability dye. Cell proliferation was assessed using CellTrace violet (CTV) according to the manufacturer’s instructions (CellTrace, Life Technologies).

### Proteomics and MS

Pellets collected from cultures of ~4 × 10^8^ CH12-F3 cells ± 10-Gy irradiation and 1-h recovery, were lysed in BLB (benzonase lysis buffer: 20 mM HEPES pH 7.9, 40 mM KCl, 2 mM MgCl_2_, 100 mM MEM, 10% glycerol, 0.5% NP-40, 50 U per ml benzonase (Novagen), 0.05% (v/v) phosphatase inhibitors (Sigma-Aldrich) and protease inhibitors (Complete EDTA-free, Roche)) and were incubated on ice for 30 min before a second incubation with adjusted salt (450 mM KCl). Flag–REV7 or control complexes were isolated from clarified lysates, following their dilution in NSB (no-salt buffer: 20 mM HEPES pH 7.9, 10% glycerol, 0.5 mM DTT, 0.5 mM EDTA, 0.05% (v/v) phosphatase inhibitors (Sigma-Aldrich) and protease inhibitors (Roche)) to a final salt concentration of 125 mM. TwinStrep–HA–SHLD1/3 complexes were immunopurified on MagStrep-XT magnetic beads (IBA), washed extensively in wash buffer (BLB supplemented with 100 mM KCl and 0.1% NP-40) and eluted with 50 μM biotin and snap-frozen for downstream proteomics. For western blot analysis, MagStep XT beads were then mixed with Laemmli buffer and boiled at 95 °C for 5 min before loading on SDS–PAGE gels. Protein samples were fractionated on NuPAGE 4–12% 1.0 mM Bis-Tris polyacrylamide gels (Life Technologies, NP0322) before transferring to 0.45-μm nitrocellulose membranes (GE Healthcare, 10600003). After transfer, membranes with blocked with 5% milk in PBST for at least 30 min and then incubated overnight with primary antibody in PBST supplemented with 0.03% NaN_3_ and 3% BSA. Primary antibodies used for western blot in this study included rabbit anti-53BP1 (1:2,500; Novus Biological, NB100-304), rabbit anti-SHLD1/2/3 (1:250; in-house), mouse anti-REV7 (1:500; BD, 612266), mouse anti-STN1 (1:500; Santa Cruz, sc-376450), mouse anti-tubulin (1:10,000; Sigma, 00020911) and rabbit anti-phospho-Chk1 (Ser345, 1:500; Cell Signaling, 2348). Horseradish peroxidase (HRP)-conjugated goat anti-rabbit IgG (Vector Laboratories, PI-1000) and HRP-conjugated horse anti-mouse IgG (Vector Laboratories, PI-2000) secondary antibodies were used and membranes developed with Clarity Western enhanced chemiluminescence substrate (Bio-Rad, 170-5061) or SuperSignal West Pico PLUS (Thermo Fisher) and imaged using a Gel Doc XR System (Bio-Rad). Images were then opened and exported for publication using Image Lab.

TwinStrep peptide eluted complexes were solubilized in 4% (w/v) SDS and 50 mM TEAB, reduced and alkylated using DTT and iodoacetamide and digested with trypsin on S-Trap microcolumns as per the manufacturer’s instructions. Samples were analyzed on an LC–MS platform (Ultimate 3000 nHPLC and Q-Exactive HFX MS instrument). Peptides were separated on an 50-cm EASY-Spray column with a gradient of 3–35% acetonitrile in 5% DMSO and 0.1% formic acid at 250 nl min^−1^. MS1 spectra were acquired at a resolution of 60,000 at 200 *m*/*z*. Up to the 15 most abundant precursor ions were selected for subsequent MS/MS analysis after ion isolation with a mass window of 1.6 Th. Peptides were fragmented by HCD with 27% collision energy. Raw files were analyzed by Progenesis QI (version 3, Waters) for spectral label-free quantitation (LFQ) with default parameters (top-three quantitation mode). Proteins were identified with PEAKS 8.0 (Bioinformatics Solutions) using standard parameters and the UniProt mouse reviewed proteome (retrieved January 23, 2020). The peptide and protein false discovery rate was set to 1%.

### Cell lines, cell culture and CRISPR–Cas9 editing

All CH12-F3 cell lines were cultured in RPMI supplemented with 5% NCTC-109 medium, 10% FCS, 100 U per ml penicillin, 100 ng ml^−1^ streptomycin and 2 mM l-glutamine at 37 °C with 5% CO_2_ under ambient oxygen conditions. *Rev7*^*−*/*−*^, *c20orf196*^*−*/*−*^ (*Shld1*^*−*/*−*^) and *Flj26957*^*−*/*−*^ (*Shld3*^*−*/*−*^) CH12-F3 cells were generated using CRISPR–Cas9 as previously described^11^. Complemented cell lines were generated by lentivirus-mediated transduction, using viral supernatants collected from 293T cells cotransfected with third-generation packaging vectors and a pLenti-PGK-TwinStrep-Flag-HA-PURO-DEST vector containing cloned transgene inserts. Typically, cells were spinoculated with viral supernatants supplemented with polybrene (8 μg ml^−1^) and HEPES (20 mM) (1,500 r.p.m., 90 min at 25 °C). Stable cell lines were subsequently selected and maintained in the presence of puromycin (1 μg ml^−1^). To stimulate CSR to IgA, CH12-F3 cells were stimulated with agonist anti-CD40 antibody (0.5 μg ml^−1^; Miltenyi Biotec; FGK45.5), mouse IL-4 (5 ng μl^−1^; R&D Systems) and transforming growth factor-β1 (2.5 ng μl^−1^; R&D Systems). Cell-surface IgA expression was determined by flow cytometric staining with anti-mouse IgA–PE antibody (Cambridge Bioscience; 1040-09).

E12.5 MEFs prepared and SV40 LargeT-immortalized by standard procedures were used in all experiments unless otherwise indicated. All MEF cell lines were maintained in high-glucose DMEM (Sigma-Aldrich, D6546) supplemented with 10% FBS, penicillin–streptomycin and 2 mM l-glutamine.

*BARD1*^*AID*/AID^ and *BARD1*^*AID/AID*^*53BP1*^*−/−*^ cell lines were generated as previously described^14,15^. All *BARD1*^*AID/AID*^ and derivative cell lines were maintained in high-glucose DMEM (Sigma-Aldrich, D6546) supplemented with 10% FBS, penicillin–streptomycin and 2 mM l-glutamine. Cultures were maintained at 37 °C with 5% CO_2_. *BARD1*^*AID/AID*^*SHLD2*^*−*/*−*^ and *BARD1*^*AID/AID*^*SHLD3*^*−*/*−*^ knockout cell lines were generated by CRISPR–Cas9 as previously described^14^. Gene-specific guide RNAs were integrated into pSpCas9(BB)-2A-GFP (PX458) (Addgene, 48138) and 2 μg of plasmid was electroporated into 10^6^ cells using a Lonza 4D-Nucleofector according to the manufacturer’s protocol for HCT-116 cells. Green fluorescent protein-positive cells were sorted 24 h after electroporation using a Sony SH800 cell sorter with the brightest 5% being pooled for recovery in medium containing 50% FBS for 4 days. Sorted populations were then seeded at low density and individual clones were isolated after 10 days of outgrowth. Individual clones were validated by sequencing.

The KB1P-G3 (ref. ^46^) *Brca1*^*−*/*−*^*p53*^*−*/*−*^ mouse mammary tumor cell lines were cultured in DMEM in the presence of 10% FCS and penicillin–streptomycin (Gibco) under low-oxygen conditions (3% O_2_, 5% CO_2_ at 37 °C). KB1P-G3 *Shld2*^*−/−*^ Cl.1 and 2 and KB1P-G3B1 (*Brca1-*reconstituted) cells were kindly gifted from S. Rottenberg^22^. KB1P-G3 *Shld3*^*−/−*^ cells were generated by CRISPR–Cas9 as previously described^11^.

### Survival experiments

To generate survival curves for and MEF cell lines, seeding assays were first performed on 10^4^ cells allowed to grow for 7 days. Following this, the growth medium was removed and the cells were washed briefly with PBS before the addition of crystal violet stain (0.5% crystal violet in 25% methanol). Cells were stained for 5 min, washed with double-distilled H_2_O and dried before scanning. Cell seeding numbers were calculated relative to WT cell density. Survival curves were then generated by seeding 300–400 WT or *Brca1*^+/*−*^*53bp1*^*−*/*−*^ cells or 650–750 *Brca1*^*−*/*−*^*53bp1*^*−*/*−*^ cells in triplicate for each drug concentration in a 96-well plate. Olaparib was added 24 h later to the indicated final concentrations.

Survival curves for *BARD1*^*AID*/AID^ and derivative cell lines were generated as previously described^14^. Briefly, 300/1000 (DMSO/IAA condition) *BARD1*^*AID/AID*^ cells, 300/700 *BARD1*^*AID/AID*^*53BP1*^*−*/*−*^ cells or 400/1500 *BARD1*^*AID/AID*^*SHLD2/3*^*−*/*−*^ cells, per well were seeded in the presence of doxycycline (2 μg ml^−1^) in triplicate for each drug concentration in a 96-well plate. After 24 h, IAA (1 mM) or carrier (DMSO) was added. Then, 1 h after IAA addition, olaparib was added to the indicated final concentrations.

For both cell lines, 7 days after drug addition, the medium was replaced with phenol red-free DMEM (Thermo Fisher, 21063-029) supplemented with 10% FBS, penicillin–streptomycin, 2 mM l-glutamine and 10 μg ml^−1^ resazurin (Sigma-Aldrich, R7017). Plates were then returned to the incubator for 2–4 h or until the growth medium in untreated control wells began to develop a pink color. Relative fluorescence was measured with a BMG LABTECH CLARIOstar plate reader. The mean of three technical repeats after background subtraction was taken as the value for a biological repeat and three biological repeats were performed for each experiment. All survival curves presented in this article represent the mean of three biological repeats ± s.e.m.

### Immunofluorescence

For RAD51 foci quantification in *BARD1*^*AID*/AID^ and derivative cell lines, 2 × 10^5^ cells were passed through a 70-μm mesh cell strainer and seeded on three fibronectin-coated glass coverslips (13 mm) in a single well of a six-well plate in the presence of doxycycline (2 μg ml^−1^). After 24 h, IAA was added to a final concentration of 1 mM. Cells were irradiated (5 Gy) 2 h after IAA addition and fixed in 2% PFA 2 h after irradiation. For RAD51 foci quantification in MEFs and KB1P-G3 cells, 4 × 10^5^ and 5 × 10^5^ cells, respectively, were seeded on three glass coverslips (13 mm) in a single well of a six-well plate. Cells were irradiated (5 Gy) and fixed in 2% PFA 2 h after irradiation. Staining of all fixed cells began with 15-min blocking (3% BSA and 0.1% Triton X-100 in PBS) followed by 1-h incubation with primary antibody in a humidity chamber. For experiments in which cells were treated with EdU, the Click-iT EdU cell proliferation kit (Alexa Fluor 647; Thermo Fisher, C10340) was used to label EdU-positive cells according to the manufacturer’s protocol between blocking and primary antibody incubation.

The following primary antibodies were used at the indicated concentrations: rabbit anti-RAD51 (HCT-116: 1:1,000; 70-001 BioAcademia, MEFS and KB1P-G3: 1:2,000; ab133534 Abcam or 1:1,000; anti-RAD51 rabbit serum, a gift from R. Kanaar) and mouse anti-γH2AX (1:500; 05-636 Millipore). Following primary antibody introduction, coverslips were washed three times with PBS containing 0.1% Triton X-100 before incubation with secondary antibody for 1 h in a humidity chamber. Secondary antibodies used in this study were as follows: goat anti-mouse Alexa Fluor 488 (1:500; A-11001 Invitrogen) and goat anti-rabbit Alexa Fluor 568 (1:500; A-11011 Invitrogen). Coverslips were then washed three times with PBS containing 0.1% Triton X-100, washed once with PBS and mounted on glass microscope slides using a drop of ProLong Gold antifade reagent with DAPI (Life Technologies, P36935).

Images were acquired on a Leica DMi8 wide-field microscope. CellProfiler (Broad Institute) was used for foci quantification. Images were visualized and saved in Fiji and assembled into figures in Affinity Designer.

### Cytological analysis

Metaphase spreads were prepared by standard methods. In brief, detached cells were resuspended in 75 mM KCl for 20 min before being fixed in Carnoy’s fixative. Approximately 20 μl of cell suspension was dropped onto clean slides and left to dry overnight. The cells were then stained with 0.5 μg ml^−1^ propidium iodide in PBS for 20 min and rinsed before the slides were mounted in Vectashield and DAPI (Vector Labs). The slides were analyzed blind and a minimum of 40 metaphases were acquired using an Olympus BX60 microscope for epifluorescence equipped with a Sensys charge-coupled device camera (Photometrics). Images were collected using Genus Cytovision software (Leica).

### Statistics

Prism 10 (GraphPad software) was used for graphing and statistical analysis. Relevant statistical methods for individual experiments are detailed within figure legends.

### Reporting summary

Further information on research design is available in the Nature Portfolio Reporting Summary linked to this article.

## Online content

Any methods, additional references, Nature Portfolio reporting summaries, source data, extended data, supplementary information, acknowledgements, peer review information; details of author contributions and competing interests; and statements of data and code availability are available at 10.1038/s41594-024-01381-9.

## Supplementary information

Supplementary Information Supplementary Fig. 1 and Table 1A,B.

Reporting Summary

## Source data

Source Data Fig. 1 Statistical source data.

Source Data Fig. 2 Statistical source data.

Source Data Fig. 2 Unprocessed western blots.

Source Data Fig. 3 Statistical source data.

Source Data Fig. 4 Statistical source data.

Source Data Fig. 5 Statistical source data.

Source Data Fig. 6 Statistical source data.

Source Data Extended Data Fig. 1 Statistical source data.

Source Data Extended Data Fig. 2 Statistical source data.

Source Data Extended Data Fig. 2 Unprocessed western blots.

Source Data Extended Data Fig. 4 Statistical source data.

Source Data Extended Data Fig. 5 Statistical source data.

Source Data Extended Data Fig. 6 Statistical source data.

## Data Availability

The MS proteomics data were deposited to the ProteomeXchange Consortium through the PRIDE partner repository with the dataset identifier PXD045534. Raw immunofluorescence images and .fcs files are available upon request. Source data are provided with this paper.
