# Supplementary material for: Shieldin and CST co-orchestrate DNA polymerase-dependent tailed-end joining reactions independently of 53BP1-governed repair pathway choice
Source: Nat Struct Mol Biol. 2024 Sep 3;32(1):86–97. doi: 10.1038/s41594-024-01381-9 (PMC11753365; doi:10.1038/s41594-024-01381-9)
Supplement: Supplementary file 1 — Supplementary Fig. 1 and Table 1A,B. [file 41594_2024_1381_MOESM1_ESM.pdf]

nature structural & molecular biology

Supplementary information https://doi.org/10.1038/s41594-024-01381-9

Shieldin and CST co-orchestrate DNA polymerase-dependent tailed-end joining reactions independently of 53BP1-governed repair pathway choice

In the format provided by the authors and unedited

Supplementary Data

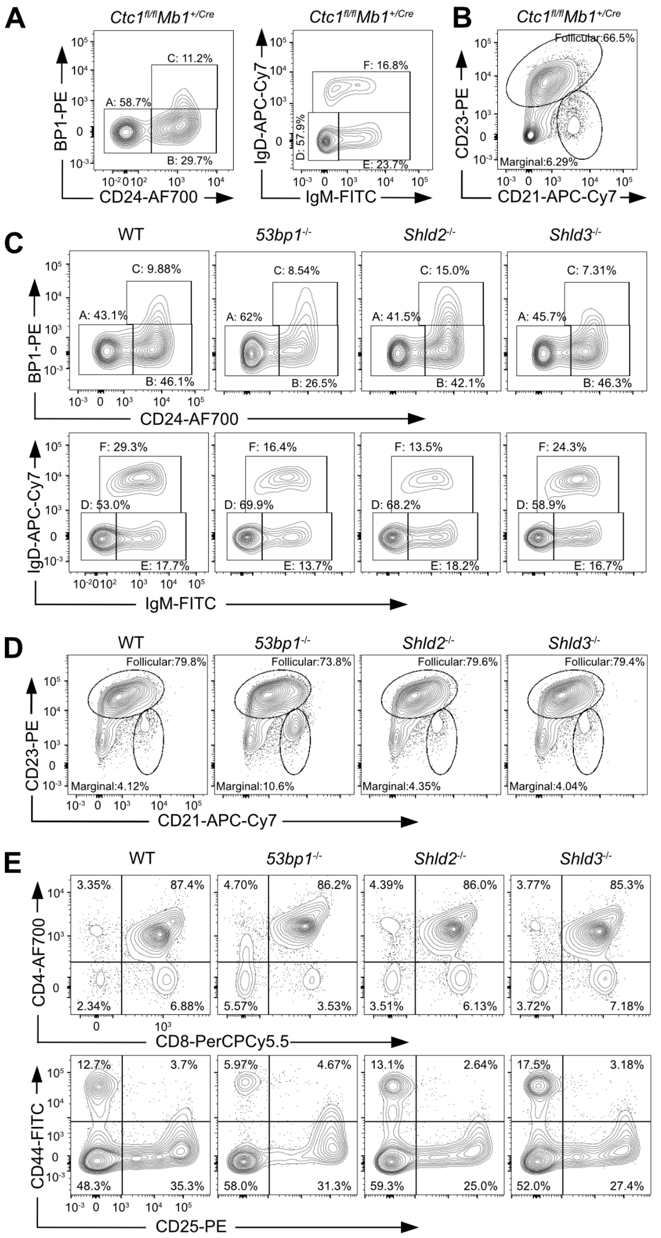

Supplementary Data Figure 1. FACS gating strategies and representative images

A) Flow cytometry analysis of B cell development in the bone marrow of *Mb1*$^{+/Cre}$, *Shld2*$^{-/-}$ ^{or} *Ctc1*$^{fl/fl}$ *Mb1*$^{+/Cre}$ *mice*; gating on B220^{+}CD43^{+} (left, Hardy fractions A, B and C) and on B220^{+}CD43^{-} (right, Hardy fractions D, E and F). Representative data; *n* = 3 experiments.

B) Flow cytometry analysis of mature b cell populations in the spleen of *Mb1*$^{+/Cre}$, *Shld2*$^{-/-}$ ^{or} *Ctc1*$^{fl/fl}$ *Mb1*$^{+/Cre}$ *mice*; gating on B220^{+}CD19^{+}. Representative data; *n* = 3 experiments.

C) Flow cytometry analysis of B cell development in the bone marrow of WT, *53bp1*$^{-/-}$, *Shld2/3*$^{-/-}$ *mice*; gating on B220^{+}CD43^{+} (top, Hardy fractions A, B and C) and on B220^{+}CD43^{-} (bottom, Hardy fractions D, E and F). Representative data; *n* > 8 experiments.

D) Flow cytometry analysis of mature b cell populations in the spleen of WT, *53bp1*$^{-/-}$, *Shld2/3*$^{-/-}$ *mice*; gating on B220^{+}CD19^{+}. Representative data; *n* > 8 experiments.

E) Flow cytometry analysis of T cell development in the thymus of WT, *53bp1*$^{-/-}$, *Shld2/3*$^{-/-}$ *mice*; gating on live cells (top) and on CD8^{-} CD4^{-} (bottom, double negative populations). Representative data; *n* >3 experiments.

Supplementary Tables

Supplementary Table 1A.

	Target	sgRNA#	Sequence	
HCT116	Shld2 (FAM35A)	sgRNA2 (5'-3')	*acctcagaatcttcctccgagg*	This study
	Shld3 (FLJ26957)	sgRNA1 (5'-3')	*cgctatcaagatttatacct*	This study

Supplementary Table 1B.

	Target gene	Clone	Genotype		
			Allele 1	Allele 2	
HCT116	Shld2 (FAM35A)	C4	+1bp at P573	+1bp at P573	This study
		C15	+2bp at P573	+1bp at P573	
	Shld3 (FLJ26957)	C5	-1bp at I39	-1bp at I39	This study
		C34	-5bp at I39	-5bp at I39	
		C42	-5bp at I39	-5bp at I39	

CRISPR–Cas9 reagents and edited cell-lines generated in this study
1A) Sequences of sgRNA used in gene editing experiments. **1B)** Genotyping of individual edited alleles in each cell-line clone, as confirmed by Sanger sequencing.
