# Supplementary material for: Shieldin and CST co-orchestrate DNA polymerase-dependent tailed-end joining reactions independently of 53BP1-governed repair pathway choice
Source: Nat Struct Mol Biol. 2024 Sep 3;32(1):86–97. doi: 10.1038/s41594-024-01381-9 (PMC11753365; doi:10.1038/s41594-024-01381-9)
Supplement: Supplementary file 2 — Reporting Summary [file 41594_2024_1381_MOESM2_ESM.pdf]

nature portfolio

Corresponding author(s): J. Ross Chapman

Last updated by author(s): Oct 17, 2023

Reporting Summary

Nature Portfolio wishes to improve the reproducibility of the work that we publish. This form provides structure for consistency and transparency in reporting. For further information on Nature Portfolio policies, see our Editorial Policies and the Editorial Policy Checklist.

Statistics

For all statistical analyses, confirm that the following items are present in the figure legend, table legend, main text, or Methods section.

n/a	Confirmed	
☐	☒	The exact sample size (*n*) for each experimental group/condition, given as a discrete number and unit of measurement
☐	☒	A statement on whether measurements were taken from distinct samples or whether the same sample was measured repeatedly
☐	☒	The statistical test(s) used AND whether they are one- or two-sided<br>*Only common tests should be described solely by name; describe more complex techniques in the Methods section.*
☒	☐	A description of all covariates tested
☐	☒	A description of any assumptions or corrections, such as tests of normality and adjustment for multiple comparisons
☐	☒	A full description of the statistical parameters including central tendency (e.g. means) or other basic estimates (e.g. regression coefficient) AND variation (e.g. standard deviation) or associated estimates of uncertainty (e.g. confidence intervals)
☐	☒	For null hypothesis testing, the test statistic (e.g. *F*, *t*, *r*) with confidence intervals, effect sizes, degrees of freedom and *P* value noted<br>*Give P values as exact values whenever suitable.*
☒	☐	For Bayesian analysis, information on the choice of priors and Markov chain Monte Carlo settings
☒	☐	For hierarchical and complex designs, identification of the appropriate level for tests and full reporting of outcomes
☒	☐	Estimates of effect sizes (e.g. Cohen's *d*, Pearson's *r*), indicating how they were calculated

Our web collection on statistics for biologists contains articles on many of the points above.

Software and code

Policy information about availability of computer code

Data collection	Mass Spectrometry: Progenesis QI 3.0, PEAKS 8.0; Gel Imaging: Image Lab v6.1.0; Flow Cytometry: Attune Nxt Software V4.2.0, FACSDiva v9.2. BMG Labtech MARS (V3.42 R6) software was used to export survival experiment data from Clariostar plate-reader. LAS X (3.7.5.24914) or Genus Cytovision v7.7 software was used for the acquisition of microscope images.
Data analysis	FlowJo v10.9.0; Perseus v2.0.11; GraphPad Prism v10.0.3 was used for presented statistical analysis, CellProfiler v4.2.5 was used for quantification of immunofluorescence images. Microscopy images were saved and manipulated using FIJI (2.9.0/1.53t). Figures were assembled using Affinity Designer 2 (2.2.0).

For manuscripts utilizing custom algorithms or software that are central to the research but not yet described in published literature, software must be made available to editors and reviewers. We strongly encourage code deposition in a community repository (e.g. GitHub). See the Nature Portfolio guidelines for submitting code & software for further information.

Data

Policy information about availability of data

All manuscripts must include a data availability statement. This statement should provide the following information, where applicable:

- Accession codes, unique identifiers, or web links for publicly available datasets
- A description of any restrictions on data availability
- For clinical datasets or third party data, please ensure that the statement adheres to our policy

The mass spectrometry proteomics data have been deposited to the ProteomeXchange Consortium via the PRIDE partner repository with the dataset identifier PXD045534 and 10.6019/PXD045534

Research involving human participants, their data, or biological material

Policy information about studies with human participants or human data. See also policy information about sex, gender (identity/presentation), and sexual orientation and race, ethnicity and racism.

Reporting on sex and gender	N/A
Reporting on race, ethnicity, or other socially relevant groupings	N/A
Population characteristics	N/A
Recruitment	N/A
Ethics oversight	N/A

Note that full information on the approval of the study protocol must also be provided in the manuscript.

Field-specific reporting

Please select the one below that is the best fit for your research. If you are not sure, read the appropriate sections before making your selection.

☒ Life sciences ☐ Behavioural & social sciences ☐ Ecological, evolutionary & environmental sciences

For a reference copy of the document with all sections, see nature.com/documents/nr-reporting-summary-flat.pdf

Life sciences study design

All studies must disclose on these points even when the disclosure is negative.

Sample size	For mouse experiments, sample sizes were determined by power calculations. We were not pursuing lower penetrance phenotypes, thus statistically significant data could typically be obtained with 4-6 mice per group (age matched mice of a single genotype), plotted with SEM and statistical significance of phenotype-specific differences determined by unpaired students t-test or two-way ANOVA.
Data exclusions	No data was excluded from the study
Replication	Following extensive optimization, biological experiments were typically performed in 2-3 biological replicates (each performed identically on different days). In the majority of cases, each biological replicate involved 3 technical replicates. To ensure reproducibility of immunization experiments, antigen-specific immune responses in each group were analyzed in two independent experiments, each yielding equivalent genotype-specific responses. For B cell lineage developmental staging, 2-3 age-matched mice per genotype were analysed in each experiment (minimum 2 experiments), each resulting in equivalent results (data presented is pooled results from all experiments). Experiments using CRISPR-Cas9 edited cell-lines: as shown throughout the manuscript, genotype specific phenotypes were revalidated across multiple independently generated knockout cells lines (n>2 per guide-RNA).
Randomization	Randomization of samples was only undertaken to remove potential biases during the scoring of chromosomal aberration during metaphase analyses. Each experiment involving mice involved comparing n>=2 mice per genotype in a single experiment, with the experiment being repeated at least twice, and typically 3 times. Age-matched mice were used as a method to control for covariates.
Blinding	Randomization of samples and blind analysis was only undertaken to remove potential biases during the scoring of chromosomal aberration during metaphase analyses.

Reporting for specific materials, systems and methods

We require information from authors about some types of materials, experimental systems and methods used in many studies. Here, indicate whether each material, system or method listed is relevant to your study. If you are not sure if a list item applies to your research, read the appropriate section before selecting a response.

Materials & experimental systems

n/a	Involved in the study
☐	☒ Antibodies
☐	☒ Eukaryotic cell lines
☒	☐ Palaeontology and archaeology
☐	☒ Animals and other organisms
☒	☐ Clinical data
☒	☐ Dual use research of concern
☒	☐ Plants

Methods

n/a	Involved in the study
☒	☐ ChIP-seq
☐	☒ Flow cytometry
☒	☐ MRI-based neuroimaging

Antibodies

Antibodies used

Antibodies used in flow cyometry studies:
anti-IgD: BioLegend 405716 Clone 11-26c.2a; 1:500,
anti-IgDa: Miltenyi Biotec 130-107-134, Clone REA484, 1:200
anti-IgM: BioLegend 406506, Clone RMM-1; 1:500
anti IgMa: BioLegend 408606, Clone MA-69, 1:200
anti-B220: BioLegend 103244, Clone RA3-6B2; 1:500
anti-BP-1: BioLegend 108308, Clone 6C3; 1:200
anti-CD19: BioLegend 115534, Clone 6D5; 1:500
anti-CD24: BD Pharmingen 562563, Clone M1/69; 1:1000
anti-CD93: BioLegend 136510, Clone AA4.1; 1:200
anti-CD23: BD Pharmingen 553139, Clone B3B4; 1:200
anti-CD43: BD Pharmingen 562865, Clone S7; 1:200
anti-CD21: BD Biosciences 563176, Clone 7G6; 1:500
anti CD44: BioLegend 103022, Clone IM7, 1:200
anti CD25: BioLegend 102008, Clone PC61, 1:500
anti CD8a: BioLegend 100732, Clone 53-6.7, 1:500
anti CD28: BioLegend 122016, Clone E18, 1:200
anti CD4: BioLegend 100430, Clone GK1.5, 1:500
antiCD3: BioLegend 100248, Clone 17A2, 1:100
anti CD71: BioLegend 113805, Clone R17217, 1:400

aanti-CD40 antibody (0.5 µg/ml; Miltenyl Biotec; FGK45.5)

anti-IgG1: BD Pharmingen 553441, Clone A85-1; 1:100
anti-IgG2b: BioLegend 406704, Clone RMG2b-1; 1:100
anti-IgG3: BD Pharmingen 553401, Clone R40-82; 1:100
anti-IgE: BioLegend 406908, Clone RME-1; 1:200
anti-IgA: Southern Biotech 1040-09; 1:250

anti-Streptavidin: Invitrogen 17-4317-82; 1:500
Zombie Aqua viability dye: BioLegend 423102; 1:200

Antibodies used in western blot studies:
anti-SHLD1/2/3: generated in house; 1:250
anti-Rev7: BD 612266, clone 14/MAD2B/Rev7, Lot 5121716; 1:500
anti-53BP1: Novus Biologicals NB100-304, Lot D-6; 1:2500
anti-STN1: Santa Cruz sc-376450, OBFC1 (E-10); 1:500
anti-phospho-Chk1 (Ser345): Cell Signaling 2348, 133D3; 1:500
anti-tubulin: Sigma 00020911, Clone TAT-1; 1:10000

Antibodies used for immunofluorescence:
rabbit anti-RAD51 (1:1000, 70-001 BioAcademia)
rabbit anti-RAD51 (1:2000, ab133534 Abcam)
polyclonal rabbit anti-RAD51 (1:1000, gifted R.Kanaar (made in-house), Erasmus MC, Rotterdam)
mouse anti-gH2AX (1:500, 05-636 Millipore)
goat anti-mouse Alexa Fluor 488 (1:500, A-11001 Invitrogen)
goat anti-rabbit Alexa Fluor 568 (1:500, A-11011 Invitrogen)
anti-Edu (Click-iT™ EdU Cell Proliferation Kit Alexa Fluor 647, C10340, Invitrogen)

Validation

All antibodies used in flow cytometry studies were validated by the manufacturers as suitable for use in flow cytometry assays against specific antigens/markers.

anti-SHLD1/2/3: validated in house using knock-out cell lines. (Figure 2d, Ext. Data Figure 2 E,F)

anti-Rev7: validated by BD and suitable for western blots; further validated in house using knock-out cell lines. https://

www.bdbiosciences.com/en-eu/products/reagents/microscopy-imaging-reagents/immunofluorescence-reagents/purified-mouse-anti-mad2b.612266.

anti-53BP1: validated by Novus Biologicals and suitable for western blot assays against mouse protein; further validated in house using knock-out cell lines. https://www.novusbio.com/products/53bp1-antibody_nb100-304.

anti-STN1: validated by Santa Cruz and suitable for western blot assays against mouse, rat and human protein. https://www.scbt.com/p/obfc1-antibody-e-10.

anti-phospho-Chk1 (Ser345): validated by Cell Signaling Technology and suitable for western blot assays against mouse, rat and human protein. https://www.cellsignal.com/products/primary-antibodies/phospho-chk1-ser345-133d3-rabbit-mab/2348.

anti-tubulin: validated by Sigma suitable for western blot assays against mouse protein. https://www.sigmaaldrich.com/GB/en/product/sigma/cb_00020911.

All antibodies used for immunofluoresence studies have been described and validated by their respective manufacturers for the purposes employed in this study.

rabbit anti-RAD51 (1:1000, 70-001 BioAcademia) https://www.abcam.com/en-gb/products/primary-antibodies/rad51-antibody-ab63801.

rabbit anti-RAD51 (1:2000, ab133534 Abcam) https://www.abcam.com/en-gb/products/primary-antibodies/rad51-antibody-epr40303-ab133534.

mouse anti-gH2AX (1:500, 05-636 Millipore) https://www.merckmillipore.com/GB/en/product/Anti-phospho-Histone-H2A.X-Ser139-Antibody-clone-JBW301,MM_NF-05-636.

Eukaryotic cell lines

Policy information about cell lines and Sex and Gender in Research

Cell line source(s)

Cell lines were either harvested from the primary tissue of indicated mice, or derived from the murine B-cell lymphoma cell CH12F3 cell-line (Francis Crick Institute Cell Services) using CRISPR-Cas9-dependent genome engineering and have been previously described and authenticated (Ghezraoui et al. 2018; DOI 10.1038/s41586-018-0362-1). The HCT116 BARD1-AID/AID cell line was generated and validated in our laboratory and has been previously described (Nakamura et al. 2019; DOI 10.1038/s41556-019-0282-9) as was the BARD1-AID/AID 53BP1-/- cell line (Becker et al. Nature 2021; DOI 10.1038/s41586-021-03776-w). BARD1-AID/AID SHLD2-/- and SHLD3-/- cell lines were derived using CRISPR-Cas9-dependent genome engineering.
Human embryonic kidney lines HEK293T (Francis Crick Institute Cell Services) were used in the generation of lentivirus.
The KB1P-G3 mouse mammary tumour cell lines were previously described (Jaspers et al. 2012; DOI 10.1158/2159-8290.CD-12-0049, Nordermeer et a. 2018; DOI: 10.1038/s41586-018-0340-7, Ghezraoui et al. 2018; DOI: 10.1038/s41586-018-0362-1).

Authentication

Genome editing on both alleles of each gene in BARD1-AID/AID cell lines were validated by amplifying the edited locus from isolated isogenic cell-line clones by PCR, and its subsequent resolution by native PAGE following random annealing (edited alleles will migrate differently to the WT parental alleles). Once editing was confirmed in this way, the edited alleles were then defined by Sanger sequencing of pooled and cloned amplicons.
HEK293T cells were used as packaging cell lines for lentivirus production and were not further authenticated.

Mycoplasma contamination

All cell lines (including HEK 293T) are tested for mycoplasma upon arrival in our laboratory.

Commonly misidentified lines (See ICLAC register)

We have checked the ICLAC register and the cell lines we used in our studies are not on the list of misidentified cell lines.

Animals and other research organisms

Policy information about studies involving animals; ARRIVE guidelines recommended for reporting animal research, and Sex and Gender in Research

Laboratory animals

All mice were generated or backcrossed (>5 generations) on a pure C57BL/6 background. Mice were aged 8-16 weeks. Mice were housed at 20-24C and 45-65% relative humidity in a 12hr light/dark cycle.

All experiments were carried out under Home Office (HO) License regulations. Both male and female animals were used in the study. The PI has a UK HO approved project licence (PP8064604), which comprised of all necessary protocols required to undertake the research outlined in the manuscript. Experiments were also undertaken in compliance with local University and ethical guidelines.

Shld2 Tm1a (Shld2tm1a(EUCOMM)Hmgu, MGI:5428631)
Flp-Cre positive mice (Tg(ACTB-Flpe)9205Dym; Jax stock 005703)
PGK-cre (B6.C-Tg(Pgk1-cre)1Lni/CrsJ; Jax stock 020811)
Shld3DEL2061-EM1
Shld3DEL2061-EM2

	Ctc1 Tm1a (Ctc1tm1a(KOMP)Wtsi/leg; MGI:4363331)<br>Trp53floxed; MGI:98834<br>Rev7 floxed; Mad2l2tm1a(EUCOMM)Wtsi/+; MGI:4432091<br>Rev3 floxed; Rev3ltm1Rsky; MGI:1337131<br>Mb1 Cre; Cd79atm1(cre)Reth; MGI:368745<br>MD4 mice; Tg(IghelMD4)4Ccg; MGI:J:109923<br>53bp1 null; Trp53bp1tm1Jc; MGI:2654201<br>Brca1null; MGI:104537<br>Shld1Δexon1 mice (C57BL/6NCrl-1110034G24Rikem1(IMPC)Mbp/Mmucd; stock 043862-UCD)
Wild animals	The study did not involve wild animals.
Reporting on sex	Both male and female animals were used in the study.
Field-collected samples	The study did not involve samples collected from the field.
Ethics oversight	All experiments were carried out under Home Office (HO) License regulations. The PI has a UK HO approved project licence (PP8064604), which comprised of all necessary protocols required to undertake the research outlined in the manuscript. Experiments were also undertaken in compliance with local University and ethical guidelines.

Note that full information on the approval of the study protocol must also be provided in the manuscript.

Plants

Seed stocks	N/A
Novel plant genotypes	N/A
Authentication	N/A

Flow Cytometry

Plots

Confirm that:

☒ The axis labels state the marker and fluorochrome used (e.g. CD4-FITC).

☒ The axis scales are clearly visible. Include numbers along axes only for bottom left plot of group (a 'group' is an analysis of identical markers).

☒ All plots are contour plots with outliers or pseudocolor plots.

☒ A numerical value for number of cells or percentage (with statistics) is provided.

Methodology

Sample preparation	Stimulated CH12F3 cell lines were washed with PBS, 2% BSA and 0.025% sodium azide (FACS buffer) and then stained for cell surface markers as per the methods section on ice for 20 min in FACS buffer. Cells were washed and resuspended in FACS buffer before acquisition.<br><br>Ex vivo splenocyte cultures, and single-cell suspensions of bone marrow and spleen were washed with FACS buffer, incubated at 4°C for 5 minutes with Mouse BD Fc block and then stained for cell surface markers on ice for 20 minutes using the reagents listed in the methods section. Cell were washed with FACS buffer and stained with Zombie Aqua Viability Dye before acquisition.<br><br>Where CFSE or CTV proliferation dyes were used, cells were washed twice in PBS with 0.1% BSA and stained with 5 µM dye for 8 minutes at room temperature in the dark. The cells were washed three times with complete growth medium and then allowed to rest for a minimum of 30 minutes at 37°C. For CH12F3 cell lines, initial CFSE signal was determined after this rest period (t=0).
Instrument	Samples were acquired on an Attune NxT (Life Technologies), BD FACSCanto or BD FACSFortessa (Becton Dickinson)
Software	Samples were analysed using FlowJo v10.9.0 (Tree Star)
Cell population abundance	At least 50, 000 cells were acquired for each condition.
Gating strategy	Bone marrow analysis: Cells were gated as nucleated (FSC-A vs SSC-A), lymphocytes (FSC-A vs SSC-A), and viable (Zombie

Gating strategy

Aqua negative). B220+CD43+ cells were then sub-gated as Hardy Fractions A (BP-1-, CD24-), B (BP1-, CD24+) and C (BP-1+, CD24+), while B220+CD43- cells were subgated as Fractions D (IgM-, IgD-), E (IgM+, IgD-) and F (IgM+, IgD+).

MD4 bone marrow analysis: Cells were gated as nucleated (FSC-A vs SSC-A), lymphocytes (FSC-A vs SSC-A), and viable (Zombie Aqua negative). B220+ cells were then subgated as Pro/pre (IgDa-IgMa-), Immature (IgDa-IgMa+) and Mature (IgDa +IgMa+).

Spleen analysis: Cells were gated as nucleated (FSC-A vs SSC-A), lymphocytes (FSC-A vs SSC-A), and viable (Zombie Aqua negative). B220+CD19+ cells were then subgated as Follicular cells (CD23+CD21+) and Marginal Zone cells (CD23-CD21high).

Thymus analysis: Cells were gated as nucleated (FSC-A vs SSC-A), lymphocytes (FSC-A vs SSC-A), and viable (Zombie Aqua negative). CD4-CD8- cells were then subgated as CD44+, CD44+CD25+, CD25+ or CD44-CD25-.

Ex vivo splenocyte class switch analysis: Cells were gated as lymphocytes (FSC-A vs SSC-A), viable (Zombie Aqua negative), proliferating cells (excluding cells which had not divided as per CTV) and IgE+, IgG1+, IgG2b+ or IgG3+.

Stimulated CH12F3 analysis: cells that successfully underwent class switch recombination were identified as IgA+.

☒ Tick this box to confirm that a figure exemplifying the gating strategy is provided in the Supplementary Information.
