# Supplementary material for: Shieldin and CST co-orchestrate DNA polymerase-dependent tailed-end joining reactions independently of 53BP1-governed repair pathway choice
Source: Nat Struct Mol Biol. 2024 Sep 3;32(1):86–97. doi: 10.1038/s41594-024-01381-9 (PMC11753365; doi:10.1038/s41594-024-01381-9)

Source Data Extended Data Figure 2E.

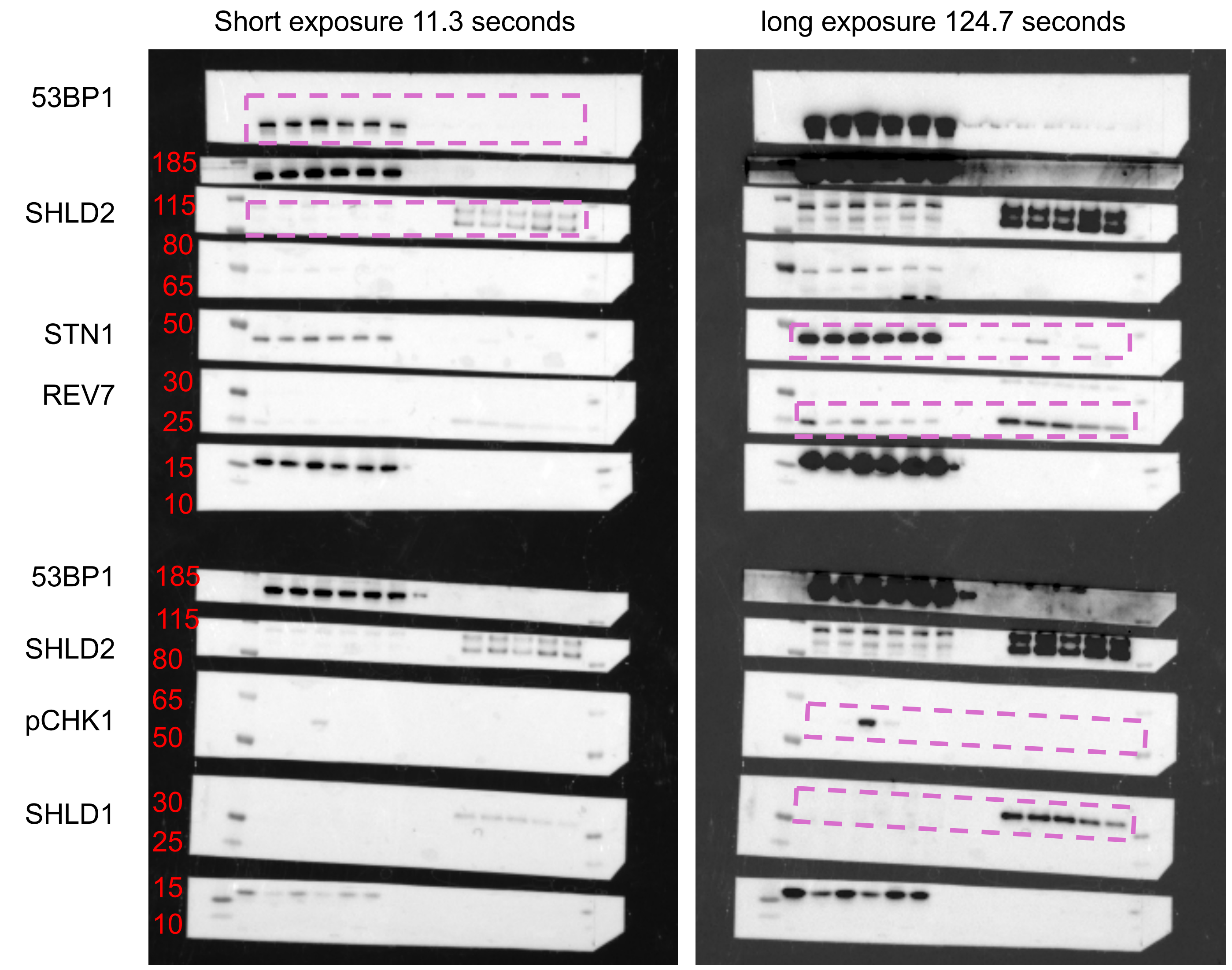

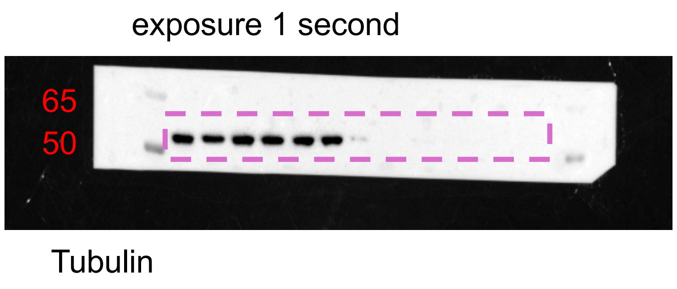

Source Data Extended Data Figure 2F.

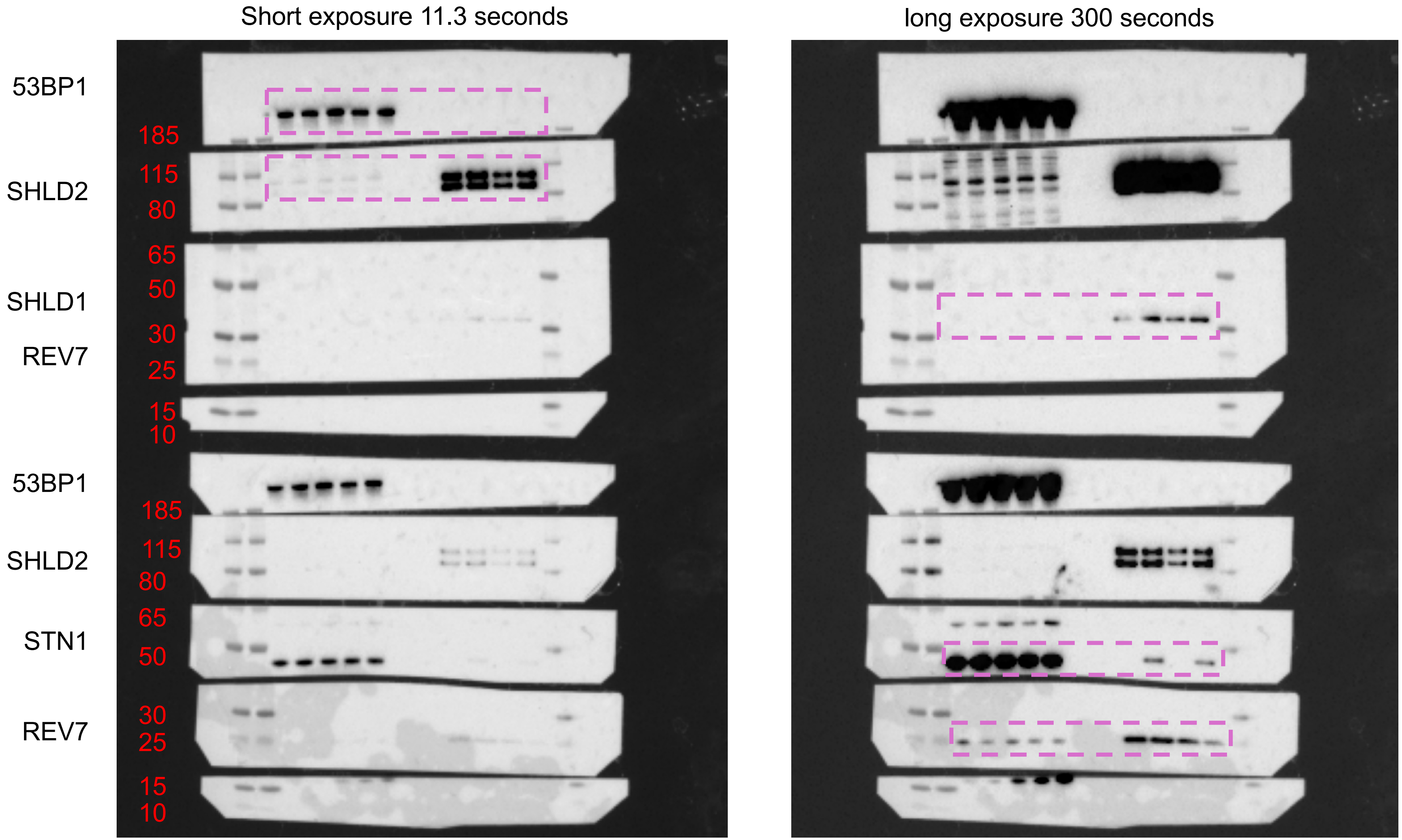

Supplement: Supplementary file 5 — Unprocessed western blots. [file 41594_2024_1381_MOESM5_ESM.pdf]
